# HP1581 (WecA)-dependent O-antigen biosynthesis governs adhesion, virulence, and biofilm dynamics in *Helicobacter pylori*

**DOI:** 10.3389/fmicb.2026.1909795

**Published:** 2026-08-19

**Authors:** Tram Thi Hong Nguyen, Mamoona Hassan, Kai-Wen Teng, Steffen Backert, Song-Wei Wang, Chung-Jung Liu, Deng-Chyang Wu, Chao-Hung Kuo, Mou-Chieh Kao

**Affiliations:** 1 Institute of Molecular Medicine, National Tsing Hua University, Hsinchu, Taiwan; 2 Division of Microbiology, Department of Biology, Friedrich-Alexander-Universität Erlangen-Nürnberg, Erlangen, Germany; 3 Division of Gastroenterology, Department of Internal Medicine, Kaohsiung Medical University Hospital, Kaohsiung, Taiwan; 4 School of Medicine, College of Medicine, Kaohsiung Medical University, Kaohsiung, Taiwan; 5 Division of Gastroenterology, Department of Internal Medicine, Kaohsiung Show Chwan Memorial Hospital, Kaohsiung, Taiwan; 6 Department of Life Science, National Tsing Hua University, Hsinchu, Taiwan; 7 School of Medicine, National Tsing Hua University, Hsinchu, Taiwan

**Keywords:** bacterial adhesion, biofilm formation, *Helicobacter pylori*, HP1581 (WecA), lipopolysaccharide, O-antigen biosynthesis, outer membrane vesicles, virulence

## Abstract

**Background:**

*Helicobacter pylori* lipopolysaccharide (LPS) contributes to bacterial fitness, immune evasion, host colonization, and virulence. The O-antigen region contains Lewis antigens and other glycans that facilitate adaptation to the gastric environment. HP1581 is predicted to encode a WecA-like UDP-*N*-acetylglucosamine-1-phosphate transferase that catalyzes the first step of O-antigen biosynthesis. However, its role in LPS assembly and *H. pylori* pathogenesis remains unclear.

**Methods:**

An *HP1581* (*wecA*) knockout mutant and a complemented strain were constructed in *H. pylori* strain 26695 to investigate the role of HP1581 in LPS biosynthesis, bacterial physiology, outer membrane vesicle (OMV) composition, host cell interactions, and virulence. Biofilm formation and swimming motility were examined using O-antigen-deficient mutants of the motile *H. pylori* G27 strain, including *HP1581* (*wecA*), *HP1206* (*wzk*), and *HP1039* (*waaL*) mutants.

**Results:**

Disruption of *HP1581* abolished O-antigen biosynthesis and eliminated Lewis X and Lewis Y antigen expression, resulting in a truncated LPS structure lacking the O-antigen region. The mutant displayed increased sensitivity to sodium dodecyl sulfate and novobiocin, elevated surface hydrophobicity and autoaggregation, and reduced survival during late-stage growth. Loss of *HP1581* significantly impaired bacterial adhesion and internalization in AGS cells and attenuated the induction of the elongated (hummingbird) phenotype. In addition, OMVs from the mutant contained substantially lower levels of CagA and VacA. In the *Galleria mellonella* infection model, the mutant exhibited reduced persistence and attenuated virulence. Furthermore, O-antigen-deficient mutants of the motile *H. pylori* G27 strain, including *HP1581* (*wecA*), *HP1206* (*wzk*), and *HP1039* (*waaL*) mutants, exhibited enhanced biofilm formation despite reduced swimming motility. Biofilm stability was found to depend primarily on extracellular proteins rather than extracellular DNA.

**Conclusion:**

HP1581 (WecA) is essential for O-antigen biosynthesis and plays critical roles in maintaining outer membrane integrity, regulating bacterial surface properties, promoting host cell interactions, modulating virulence-associated factors, and influencing biofilm development. These findings identify HP1581 (WecA)-dependent O-antigen biosynthesis as a key determinant of *H. pylori* virulence and persistence and highlight HP1581 as a potential therapeutic target.

## 1 Introduction

*Helicobacter pylori* (*H. pylori*) is a major human gastric pathogen that chronically infects approximately half of the world's population and is strongly associated with the development of chronic gastritis, peptic ulcer disease, gastric adenocarcinoma, and mucosa-associated lymphoid tissue lymphoma (Malfertheiner et al., 2023). Despite the availability of antibiotic-based eradication therapies, treatment efficacy has declined owing to the increasing prevalence of antimicrobial resistance and treatment failure (Gaddy et al., 2015). Although alternative approaches, including bacteria-targeting biomaterials, have shown promise, their clinical effectiveness remains to be fully established (Lai et al., 2022). Therefore, identifying novel bacterial targets involved in colonization and pathogenesis remains an important objective for the development of future therapeutic strategies.

Successful colonization of the gastric mucosa by *H. pylori* depends on its ability to adhere to gastric epithelial cells and mucus layers. This process is mediated through interactions between bacterial adhesins and host cell receptors, particularly fucosylated and sialylated glycoconjugates such as Lewis b (LeB) and sialyl-Lewis X (sLeX) expressed on gastric epithelial cells (Bonsor and Sundberg, 2019; Matos et al., 2021; Sijmons et al., 2024). Several outer membrane proteins, including BabA, SabA, OipA, AlpA/AlpB, and HopQ, contribute to bacterial attachment, colonization, and the efficient delivery of virulence factors such as CagA into host cells (Kao et al., 2016; Matsuo et al., 2017; Matos et al., 2021).

**Figure 1:**
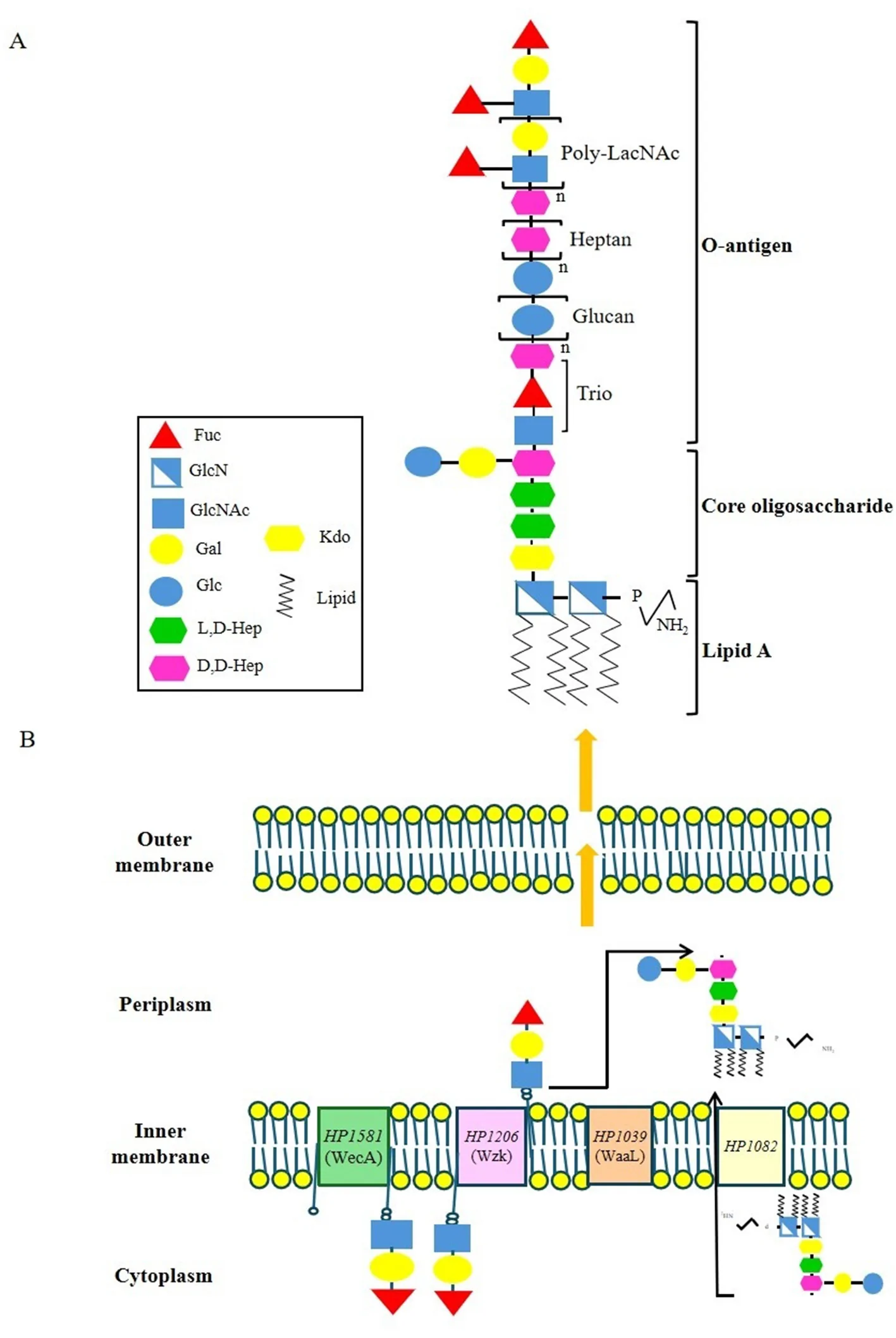
Proposed model of O-antigen biosynthesis in *Helicobacter pylori*. **(A)** Schematic representation of the LPS structure of *H. pylori* 26695, highlighting its three major domains: lipid A, the core oligosaccharide, and the O-antigen. **(B)** Proposed roles of three key enzymes involved in O-antigen biosynthesis. WecA (HP1581) initiates O-antigen assembly by transferring N-acetylglucosamine-1-phosphate (GlcNAc-1-P) from UDP-N-acetylglucosamine (UDP-GlcNAc) to the undecaprenyl phosphate (Und-P) lipid carrier. The resulting O-antigen precursor is subsequently translocated across the inner membrane by the flippase Wzk (HP1206). Finally, the O-antigen is ligated to the lipid A-core oligosaccharide by the ligase WaaL (HP1039), forming mature LPS.

Lipopolysaccharide (LPS), a major constituent of the outer membrane of Gram-negative bacteria, also plays a pivotal role in *H. pylori* colonization and persistence. In addition to maintaining outer membrane integrity and serving as a permeability barrier against harmful compounds, LPS contributes to immune evasion through molecular mimicry of host glycans (Moran, 1996; Nikaido, 2003; Delcour, 2009; Silhavy et al., 2010). Structurally, *H. pylori* LPS is nearly linear and consists of three major domains: lipid A, a core oligosaccharide, and an O-antigen polysaccharide (Figure 1A; Zhuang et al., 2024). The lipid A moiety exhibits unusually low endotoxic activity compared with that of enteric Gram-negative bacteria owing to its unique acylation pattern (Fujimoto et al., 2012; Matsuura, 2013; Li et al., 2016). The conserved core oligosaccharide is composed of 3-deoxy-D-manno-oct-2-ulsonic acid (Kdo), two LD-heptose residues, one DD-heptose residue, and a glucose-galactose branch (-Glc-Gal-DD-Hep-LD-Hep-LD-Hep-Kdo-). The O-antigen region contains a conserved trisaccharide motif, designated Trio (GlcNAc-Fuc-DD-Hep), together with α-1, 6-glucan, DD-heptan homopolymers, poly-N-acetyllactosamine (poly-LacNAc) structures, and Lewis antigen determinants (Altman et al., 2003; Nilsson et al., 2006; Zhuang et al., 2024). Notably, *H. pylori* LPS frequently expresses Lewis X (LeX) and Lewis Y (LeY) antigens that closely resemble human gastric epithelial glycoconjugates, thereby facilitating immune evasion, host adaptation, and persistent colonization of the gastric mucosa (Edwards et al., 2000; Li et al., 2016).

Bacterial surface glycoconjugates are essential for diverse biological processes, including host colonization, immune evasion, biofilm formation, and virulence. The biosynthesis of these glycoconjugates is initiated by membrane-associated phosphoglycosyl transferases (PGTs), which transfer activated sugar residues onto undecaprenyl phosphate (Und-P) lipid carriers at the cytoplasmic membrane (O'Toole et al., 2021; Durand et al., 2024). Among these enzymes, WecA and MraY belong to the polyprenyl-phosphate N-acetylhexosamine 1-phosphate transferase (P2HPT) superfamily and share conserved membrane topologies and catalytic mechanisms (Lukose et al., 2017). While MraY participates in peptidoglycan biosynthesis, WecA catalyzes the transfer of N-acetylglucosamine-1-phosphate (GlcNAc-1-P) from UDP-GlcNAc to Und-P, thereby initiating the biosynthesis of O-antigens and several other cell-envelope glycoconjugates (Al-Dabbagh et al., 2008).

Based on sequence homology and genome annotation, *HP1581* has been predicted to encode a WecA-like undecaprenyl-phosphate *N*-acetylglucosamine-1-phosphate transferase that catalyzes the first committed step of O-antigen biosynthesis by transferring GlcNAc-1-phosphate to undecaprenyl phosphate (Und-P), thereby generating UndPP-GlcNAc on the cytoplasmic face of the inner membrane (Figure 1B; Hug et al., 2010; Jia et al., 2017). Subsequent glycosyltransferases and fucosyltransferases elongate the glycan chain, which is then translocated across the inner membrane by the flippase Wzk (HP1206) and ligated to the lipid A-core oligosaccharide by WaaL (HP1039) to generate mature LPS (Hug et al., 2010; Kalynych et al., 2014; Zhao et al., 2014). However, although HP1581 has been proposed to function as the initiating glycosyltransferase in O-antigen biosynthesis, its biological role has not been fully validated in *H. pylori*. While several enzymes involved in the downstream steps of O-antigen biosynthesis have been functionally characterized, the contribution of HP1581 to *H. pylori* physiology and pathogenesis remains poorly understood.

Previous studies have shown that disruption of genes involved in LPS core oligosaccharide and fucose biosynthesis compromises bacterial fitness, host-cell interactions, and virulence in *H. pylori* (Chang et al., 2011; Yu et al., 2016; Chiu et al., 2021; Liu et al., 2022a; Nguyen et al., 2025). These LPS-deficient mutants also exhibit impaired adhesion to and invasion of gastric epithelial cells, highlighting the importance of intact LPS structures in host colonization and pathogenesis. Despite these advances, the biological significance of WecA, which catalyzes the initial step of O-antigen biosynthesis, remains poorly understood. In particular, its contributions to LPS assembly, virulence factor delivery, bacterial fitness, and biofilm development have not been systematically investigated.

To address this knowledge gap, we constructed an *HP1581* knockout mutant and a corresponding complemented strain in the well-characterized *H. pylori* strain 26695 to characterize the biological function of HP1581. Because strain 26695 is non-motile and exhibits limited biofilm-forming ability, whereas strain G27 is motile, flagellated, and readily forms biofilms, biofilm-related analyses were performed using O-antigen-deficient mutants derived from the G27 background (Giacomucci et al., 2019). Our results demonstrate that HP1581 is indispensable for O-antigen biosynthesis and plays critical roles in maintaining outer membrane integrity, regulating surface hydrophobicity and autoaggregation, promoting host-cell adhesion and virulence, influencing outer membrane vesicle composition, and modulating biofilm formation. Collectively, these findings provide new insights into the multifaceted biological functions of WecA and establish this enzyme as a key determinant of *H. pylori* fitness and pathogenesis. Moreover, HP1581 represents a potential therapeutic target for the development of novel anti-*H. pylori* strategies, although the consequences of disrupting O-antigen biosynthesis on biofilm formation warrant careful consideration.

## 2 Materials and methods

### 2.1 Bacterial strains, plasmids, and growth conditions

The bacterial strains and plasmids used in this study are listed in Table 1. *H. pylori* strain 26695 (ATCC 700392; CagA^+^ VacA^+^; Tomb et al., 1997) and strain G27 (CagA^+^ VacA^+^; Baltrus et al., 2009), together with their corresponding mutant derivatives, were routinely cultured on Columbia agar plates supplemented with 5% (w/v) sheep blood under microaerophilic conditions (5% O_2_, 10% CO_2_, and 85% N_2_) at 37 °C for 48–72 h. For liquid culture, bacteria were grown in Brucella broth (BD Biosciences, Franklin Lakes, NJ, USA) supplemented with 10% fetal bovine serum (FBS; Biological Industries, Kibbutz Beit Haemek, Israel) and 1% IsoVitalex enrichment (Taiwan Prepared Media, Taipei, Taiwan). Cultures were incubated at 37 °C under microaerophilic conditions with shaking at 120 rpm using a horizontal shaker incubator (LM-570RD, Yihder Co., New Taipei City, Taiwan) for the indicated time periods. *Escherichia coli* DH5α (Thermo Fisher Scientific, Waltham, MA, USA) was used for plasmid propagation and maintained in Luria–Bertani (LB) broth or on LB agar plates supplemented with appropriate antibiotics when required.

**Table 1:** Bacterial strains and plasmids used in this study.

	Strain/plasmid	Characteristic and marker^a^	Source
Strain	*E. coli* DH5α	The host for the construction of pGEM-T-*gene*::Kan^r^ clone	Invitrogen, Carlsbad, CA, USA
	*H. pylori* 26695 WT	*H. pylori* whole-genome sequencing strain, isolated from the stomach of a patient with gastritis	ATCC^b^ 700392
	*H. pylori* 26695 KO 1581	*H. pylori* 26695 strain with a kanamycin-resistant cassette in *HP1581*; Kan^r^	This study
	*H. pylori* 26695 Com 1581	KO 1581 with *HP1581* insertion in *HP0954* (RdxA); Met^r^, Kan^r^	This study
	*H. pylori* 26695 G27 WT	*H. pylori* whole-genome sequencing strain, isolated from an endoscopy patient from Grosseto Hospital (Tuscany, Italy)	Baltrus et al., 2009
	*H. pylori* G27 KO 1581/HPG27_1518	*H. pylori* G27 strain with a kanamycin-resistant cassette in *HPG27_1518*; Kan^r^	This study
	*H. pylori* G27 KO 1206/HPG27_1153	*H. pylori* G27 strain with a chloramphenicol-resistant cassette in *HPG27_1153*; Cm^r^	This study
	*H. pylori* G27 KO 1039/HPG27_389	*H. pylori* G27 strain with a chloramphenicol-resistant cassette in *HPG27_389*; Cm^r^	This study
Plasmid	pGEM-T	TA cloning vector; Amp^r^	Promega, Madison, WI, USA
	pGEM-T-HP1581	pGEM-T with *HP1581* DNA fragment; Amp^r^	This study
	pGEM-T-RdxA_L_-P*_*HP*1581_*-*HP1581*-T7_ter_-RdxA_R_	pGEM-T with *HP1581* insertion in *HP0954* (RdxA); Met^r^, Kan^r^	This study
	pGEM-T-HPG27_1518	pGEM-T with *HPG27_1518* DNA fragment; Amp^r^	This study
	pGEM-T-HPG27_1153 PGEM-T-HPG27_389	pGEM-T with *HPG27_1153* DNA fragment; Amp^r^ pGEM-T with *HPG27_389* DNA fragment; Amp^r^	This study This study

^a^Amp^r^, ampicillin-resistant; Cm^r^, chloramphenicol-resistant; Kan^r^, kanamycin-resistant; Met^r^, metronidazole-resistant. ^b^ATCC, American Type Culture Collection.

### 2.2 Construction of the targeted gene knockout and the *HP1581* complemented mutants in *H. pylori*

Gene knockout mutants were generated by allelic exchange mutagenesis using a splicing-by-overlap-extension (SOEing) polymerase chain reaction (PCR) strategy as previously described (Noto and Peek, 2012; Rondina et al., 2020). Briefly, the upstream and downstream flanking regions of each target gene were amplified using mutagenic primers containing *BamHI* restriction sites, generating overlapping DNA fragments. The amplified fragments were fused by overlap-extension PCR and cloned into the pGEM-T Easy vector (Promega, Madison, WI, USA) using T4 DNA ligase (New England Biolabs, Ipswich, MA, USA). A kanamycin- or chloramphenicol-resistance cassette was subsequently inserted into the *BamHI* site to disrupt the target gene. The resulting recombinant plasmids were transformed into *E. coli* DH5α for propagation and sequence verification. Purified plasmids were then introduced into *H. pylori* by natural transformation, and recombinant mutants were selected on blood agar plates supplemented with the appropriate antibiotics. Target genes analyzed in this study included *HP1581, HPG27_1518, HPG27_1153*, and *HPG27_389*. Primers used for mutant construction are listed in Table 2. For genetic complementation, a DNA fragment containing the native *HP1581* promoter region (approximately 370 bp upstream of the translational start site) and the complete *HP1581* open reading frame (1,011 bp) was amplified from *H. pylori* 26695 genomic DNA by SOEing PCR using the primers listed in Table 2. The resulting PCR product was cloned into the pJET1.2/blunt vector (Thermo Fisher Scientific) and propagated in *E. coli* DH5α. Following digestion with *NcoI* and *KpnI*, the *HP1581* expression cassette was inserted into a modified pGEM-T-based complementation vector containing homologous regions flanking the *rdxA* (*HP0954*) locus (pGEM-T-RdxA_L_-P_*HP*1581_-*HP1581*-T7_ter_-RdxA_R_). The complementation construct was introduced into the *HP1581* knockout mutant by natural transformation, and complemented strains were selected on blood agar plates containing kanamycin and metronidazole. Correct chromosomal integration and gene replacement were confirmed by PCR amplification using primers flanking the target loci, followed by DNA sequencing. Restriction enzymes were purchased from New England Biolabs (Ipswich, MA, USA), while plasmid purification and gel extraction kits were obtained from Biokit (Miaoli, Taiwan). Antibiotics were purchased from Sigma-Aldrich (St. Louis, MO, USA).

**Table 2:** Sequences and restriction sites of the primers used in this study.

Primer	Restriction enzyme site	Sequence (5^′^ → 3^′^)^e^
KO1581F1^a^	–	CATCATGCAAGAACCCCACG
KO1581R1^a^	*Bam*HI	CAAGGGATCCCACCATGTAAGCTAAT
KO1581F2^a^	*Bam*HI	GGTGGGATCCCTTGGGTTTATGGTGT
KO1581R2^a^	–	GCACAAACTTGCCTGTTCAAATAAGC
Com1581F1^b^	*Nco*I	AGTACCATGGTTGACTTGGATTTC
Com1581R1^b^	–	CGCCTATGACTAAGATTTTTTTCATATCGTAACTCC
Com1581F2^b^	–	GGAGTTACGATATGAAAAAAATCTTAGTCATAGGCG
Com1581R2^b^	*Kpn*I	AGTAGGTACCTCAATCATTGC
KO1206F1^c^	–	CCTGCTTGTGCTGATGGCTG
KO1206R1^c^	*Bam*HI	TTTTGGATCCCTTGATGAGAACGGTG
KO1206F2^c^	*Bam*HI	CAAGGGATCCAAAAAGGGTGAGATGG
KO1206R2^c^	–	TTCTGACCTCTTTGAATGGTGAGG
KO1039F1^d^	–	TCATTCCAGTCGTTTAGGGC
KO1039R1^d^	*Bam*HI	GAAAGGATCCGTTTGGCTATCATG
KO1039F2^d^	*Bam*HI	AAACGGATCCTTTCCGCTACAATT
KO1039R2^d^	–	TCCCCACATACAAAGCGCTC

^a^Primers used in the construction of the HP1581 knockout mutant. ^b^Primers used in the construction of the HP1581 complemented strain. ^c^Primers used in the construction of the HP12061 complemented strain. ^d^Primers used in the construction of the HP1039 complemented strain. ^e^The sequences of restriction enzyme sites are underlined.

### 2.3 Bioinformatic analyses of HP1581

Genomic and protein sequence information for HP1581 from *H. pylori* strain 26695 was retrieved from the Kyoto Encyclopedia of Genes and Genomes (KEGG) and UniProt databases. The genomic organization of *HP1581* and its neighboring genes was examined using the KEGG genome database. Sequence similarity searches were performed using the Basic Local Alignment Search Tool (BLAST; https://blast.ncbi.nlm.nih.gov/Blast.cgi). Conserved protein domains were identified using the NCBI Conserved Domain Database (CDD). Transmembrane helices were predicted using the TMPred server (https://embnet.vital-it.ch/software/TMPRED_form.html). Homologous WecA protein sequences from representative Gram-negative bacteria were aligned using Sequence Manipulation Suite 3 (SMS3), version 0.0.1 (https://stothardresearch.ca/sequence-manipulation-suite/#tool=multiple-align-protein).

### 2.4 LPS isolation and analysis

LPS was isolated from *H. pylori* strains using a modified hot phenol extraction method. Briefly, bacteria were grown on sheep blood agar plates for 48 h, harvested, and resuspended in 600–800 μL of lysis buffer containing 1 M Tris-HCl, 1 M NaCl, 0.5 M EDTA, and 10% SDS. Protein concentrations were determined using a bicinchoninic acid (BCA) protein assay kit (Thermo Fisher Scientific), and equivalent amounts of bacterial lysates, normalized according to total protein content, were used for subsequent LPS extraction. Whole-cell lysates were treated with 15 μL of proteinase K (20 mg/mL; Sigma-Aldrich), adjusted to a final volume of 150 μL with deionized water, and incubated at 60 °C for 2 h to remove contaminating proteins. LPS was extracted by adding an equal volume of 90% hot phenol prewarmed to 70 °C, followed by incubation at 70 °C for 15 min with gentle inversion every 5 min. Samples were then cooled on ice for 10 min and centrifuged at 10,000 × g for 10 min at 4 °C. The aqueous phase containing LPS was carefully collected and used for subsequent analyses. For LPS profile analysis, purified LPS samples were separated on 15% Tricine-sodium dodecyl sulfate polyacrylamide gel electrophoresis (SDS-PAGE) gels and visualized by silver staining. Following electrophoresis, gels were fixed overnight in fixing solution (25% isopropanol and 7% acetic acid), oxidized for 10 min in oxidation solution (27% ethanol, 0.7% periodic acid, and 0.3% acetic acid), and stained with silver nitrate solution containing 0.1 N NaOH, ammonium hydroxide, and 20% silver nitrate. After extensive washing with deionized water, LPS bands were developed in a solution containing 0.074% formaldehyde and 0.05% citric acid until clear band patterns became visible. To determine Lewis antigen expression, purified LPS samples were separated by 15% SDS-PAGE and transferred onto 0.45-μm nitrocellulose membranes (Millipore, Billerica, MA, USA). Membranes were blocked with 5% skim milk in phosphate-buffered saline (PBS) for 1 h and incubated overnight at 4 °C with mouse monoclonal antibodies against Lewis X (anti-Lewis X; 1:1,000; ab3558, Abcam, Cambridge, UK) or Lewis Y (anti-Lewis Y; 1:1,000; ab3359, Abcam). After washing, membranes were incubated with horseradish peroxidase (HRP)-conjugated goat anti-mouse IgM antibody (1:10,000; Thermo Fisher Scientific) for 1 h at room temperature. Immunoreactive signals were detected using an ImageQuant LAS 4,000 mini imaging system (GE Healthcare, Chicago, IL, USA).

### 2.5 Growth curve analysis and susceptibility to SDS and novobiocin

To evaluate the effects of *HP1581* disruption on bacterial growth and outer membrane integrity, growth kinetics and susceptibility to the detergent SDS and the hydrophobic antibiotic novobiocin were assessed. Because SDS and novobiocin are commonly used probes of outer membrane permeability in Gram-negative bacteria, increased susceptibility to these agents was interpreted as an indicator of compromised outer membrane integrity (Nikaido, 2003; Delcour, 2009). For growth curve analysis, *H. pylori* strains were harvested from sheep blood agar plates after 48 h of incubation and inoculated into Brucella broth supplemented with 10% FBS and 1% IsoVitalex. Following incubation under microaerophilic conditions at 37 °C for 16 h, bacterial cultures were diluted to an optical density at 600 nm (OD_600_) of 0.05 and further cultivated in fresh medium under the same conditions, with shaking at 140 rpm. Bacterial growth was monitored for up to 84 h by measuring the OD_600_ at the indicated time points. To assess susceptibility to SDS and novobiocin, bacterial cultures were grown in Brucella broth under microaerophilic conditions for 36 h and adjusted to an OD_600_ of 0.6. Aliquots (1 mL) were transferred into 24-well plates containing the indicated concentrations of SDS or novobiocin and incubated under microaerophilic conditions at 37 °C with shaking at 140 rpm for 72 h. Bacterial growth was subsequently quantified by measuring OD_600_, and the survival rate was calculated as follows: Survival rate (%) = (OD_600_ of treated culture/OD_600_ of untreated control culture) × 100.

### 2.6 Surface hydrophobicity and autoaggregation assays

Surface hydrophobicity and autoaggregation assays were performed as previously described with minor modifications (Nguyen et al., 2025). Briefly, *H. pylori* strains were scraped from sheep blood agar plates and cultured in Brucella broth under microaerophilic conditions at 37 °C with shaking at 140 rpm for 48 h. For the surface hydrophobicity assay, bacterial cells were harvested by centrifugation (4,000 × g, 10 min, 4 °C), resuspended in phosphate-urea-magnesium sulfate (PUM) buffer (22.2 g/L K_2_HPO_4_·3H_2_O, 7.26 g/L KH_2_PO_4_, 1.8 g/L urea, and 0.02 g/L MgSO_4_·7H_2_O; pH 7.1), and adjusted to an OD_600_ of 1.0 in a final volume of 5 mL. Subsequently, 400 μL of *n*-hexadecane was added to each bacterial suspension. The mixtures were vortexed vigorously for 2 min and then allowed to stand undisturbed at room temperature for up to 24 h to permit phase separation. The absorbance of the aqueous phase was measured at 600 nm immediately after mixing (A_0_) and at the indicated time points thereafter (A_t_). The hydrophobicity index was calculated using the following equation: Hydrophobicity index = [(A_0_ – A_t_)/A_0_]. Higher hydrophobicity index values indicate greater affinity of bacterial cells for the hydrocarbon phase and increased cell-surface hydrophobicity.

For the autoaggregation assay, bacterial cells were harvested by centrifugation (4,000 × g, 10 min, 4 °C), resuspended in 1 × PBS, and adjusted to an OD_600_ of 1.0 in a final volume of 6 mL. Bacterial suspensions were transferred to sterile tubes and incubated statically at room temperature for up to 24 h. At the indicated time points, the upper aqueous phase was carefully collected without disturbing the settled aggregates, and the absorbance at 600 nm was recorded. Autoaggregation was calculated according to the following equation: Autoaggregation rate (%) = [(A_0_ – At)/A_0_] × 100. Higher autoaggregation values indicate a greater tendency of bacterial cells to aggregate and sediment. To verify that the decrease in optical density observed during the autoaggregation assay was attributable to bacterial cell aggregation rather than non-specific sedimentation, 10 μL aliquots were carefully collected from the undisturbed bottom phase of the bacterial suspensions after static incubation and placed onto glass microscope slides. Samples were visualized using an Olympus IX71 inverted microscope (Olympus, Tokyo, Japan) at 200 × magnification, and representative images were acquired to assess aggregate formation.

### 2.7 Isolation and analysis of whole-cell lysate and outer membrane vesicles (OMVs)

Whole-cell lysates and OMVs were prepared as previously described with minor modifications (Wai et al., 1995; Chiu et al., 2021; Liu et al., 2022). *H. pylori* strains were cultured in Brucella broth supplemented with 10% FBS and 1% IsoVitalex under microaerophilic conditions at 37 °C with shaking at 140 rpm for 48 h for whole-cell lysate preparation and 60 h for OMV isolation. For whole-cell lysate preparation, bacterial cultures were harvested by centrifugation (4,000 × g, 10 min, 4 °C), resuspended in 400–600 μL of lysis buffer (50 mM Tris-HCl [pH 7.5], 150 mM NaCl, 1 mM EDTA, and 1% SDS), and incubated on ice for 30 min. Cell debris was removed by centrifugation (10,000 × g, 10 min, 4 °C), and the resulting supernatants containing soluble proteins were collected as whole-cell lysates. Protein concentrations were determined using a BCA protein assay kit (Thermo Fisher Scientific), and equivalent amounts of lysates, normalized according to total protein content, were used for subsequent analyses. For OMV isolation, bacterial cultures were centrifuged (4,000 × g, 10 min, 4 °C), and the resulting culture supernatants were passed through 0.45-μm membrane filters to remove residual bacterial cells and large debris. The filtered supernatants were subsequently subjected to ultracentrifugation at 200,000 × g for 120 min at 4 °C using a CS150NX micro-ultracentrifuge (Hitachi Instruments Inc., Tokyo, Japan). The resulting OMV pellets were resuspended in 20 mM Tris-HCl (pH 7.4). Protein concentrations of OMV preparations were quantified using the BCA assay. For comparative analyses, OMV samples were normalized to total protein content, and equivalent amounts of OMV protein were used for SDS-PAGE, Coomassie Brilliant Blue staining, and immunoblotting. Proteins were separated on 10% SDS-PAGE gels and either visualized by staining with 0.25% Coomassie Brilliant Blue R-250 (Sigma-Aldrich) or transferred onto 0.45-μm nitrocellulose membranes for immunoblotting. Membranes were blocked with 5% skim milk in PBS for 1 h at room temperature and incubated overnight at 4 °C with the following primary antibodies: mouse anti-CagA (1:1,000; sc-28368, Santa Cruz Biotechnology, Dallas, TX, USA), rabbit anti-VacA (1:3,000; HHP-5013-9, Austral Biologicals, San Ramon, CA, USA), or rabbit anti-UreA (1:10,000; PA5-117505, Thermo Fisher Scientific). After washing, membranes were incubated with HRP-conjugated goat anti-mouse or goat anti-rabbit secondary antibodies (1:10,000; Thermo Fisher Scientific) for 1 h at room temperature. Immunoreactive signals were detected using an ImageQuant LAS 4,000 mini imaging system (GE Healthcare).

### 2.8 Gastric cell culture and bacterial infection assay

Human gastric adenocarcinoma AGS cells (ATCC CRL-1739) were maintained in Ham's F-12 medium (Sigma-Aldrich) supplemented with 10% FBS and 1% penicillin–streptomycin (Gibco, Thermo Fisher Scientific) at 37 °C in a humidified atmosphere containing 5% CO_2_. For bacterial infection experiments, AGS cells were seeded at 2 × 10^5^ cells per well in 12-well plates and cultured for 24 h to reach approximately 80% confluency. Prior to infection, cells were washed once with PBS and incubated in antibiotic-free Ham's F-12 medium. *H. pylori* strains grown to the stationary phase were added to AGS cells at a multiplicity of infection (MOI) of 100 and co-cultured for 6 h in Ham's F-12 medium supplemented with 10% FBS. Following infection, cellular morphological changes were examined using an Olympus IX71 inverted microscope (Olympus) at 200 × magnification, and representative images were captured.

### 2.9 Adhesion assay

The adhesion assay was performed as described above, following the bacterial infection procedure. After 6 h of co-culture with *H. pylori* strains, AGS cells were washed three times with PBS to remove non-adherent bacteria. The infected cells were then lysed with 1 mL of PBS containing 0.1% saponin to release cell-associated bacteria. The resulting lysates were serially diluted in PBS and plated onto sheep blood agar plates. Following incubation under microaerophilic conditions at 37 °C for 72–96 h, viable bacteria were quantified by colony-forming unit (CFU) enumeration. Adhesion efficiency was expressed as the number of cell-associated bacteria recovered from infected AGS cells.

### 2.10 Internalization assay

The internalization assay was performed as described above, following the bacterial infection procedure. After 6 h of co-culture with *H. pylori* strains, AGS cells were washed with PBS to remove non-internalized bacteria and subsequently incubated in Ham's F-12 medium supplemented with 10% FBS and 100 μg/mL gentamicin for 1 h to eliminate any remaining extracellular bacteria. Following gentamicin treatment, the cells were washed with PBS and further incubated in Ham's F-12 medium containing 10% FBS and 10 μg/mL gentamicin for an additional 24 h under standard cell culture conditions. The infected cells were then lysed with 0.5% saponin in PBS for 5 min to release intracellular bacteria. Cell lysates were serially diluted in PBS and plated onto sheep blood agar plates. After incubation under microaerophilic conditions at 37 °C for 72–96 h, viable intracellular bacteria were quantified by CFU enumeration. Internalization efficiency was expressed as the number of intracellular bacteria recovered from 2 × 10^5^ infected AGS cells.

### 2.11 Analysis of *H. pylori*-induced morphological changes in AGS cells

To evaluate the ability of *H. pylori* strains to induce morphological alterations in gastric epithelial cells, AGS cells were seeded at 2 × 10^5^ cells per 6 cm plate and cultured for 24 h. Cells were then infected with *H. pylori* strains at an MOI of 100 and incubated for 6 h at 37 °C in a humidified atmosphere containing 5% CO_2_. Following infection, cellular morphology was examined using an Olympus IX71 inverted microscope (Olympus) at 200 × magnification. Cells exhibiting the characteristic elongated morphology induced by *H. pylori* infection were classified as elongation-positive cells. Representative images were captured for qualitative comparison among strains. Microscopic fields were randomly selected from each plate for quantification. The proportion of AGS cells with elongated morphology was quantified by analyzing 10 distinct microscopic fields, each measuring 0.25 mm^2^. The percentage of elongation-positive cells was calculated as follows: Elongation phenotype (%) = (number of elongation-positive cells/total number of AGS cells) × 100.

### 2.12 Assessment of virulence using the *Galleria mellonella* (*G. mellonella*) infection model

The virulence of *H. pylori* strains was evaluated using the *G. mellonella* larval infection model as previously described with minor modifications (Askoura and Stintzi, 2017; Jorjão et al., 2018; Nguyen et al., 2025). Ethical approval was not required for the use of *G. mellonella* larvae. Final-instar larvae weighing approximately 0.1–0.3 g and measuring 2.5–3 cm in length were maintained in the dark at 10–15 °C prior to experimentation. Only healthy larvae displaying a uniform cream-colored appearance and no visible signs of melanization or physical damage were selected for infection studies. *H. pylori* strains were harvested from sheep blood agar plates, resuspended in PBS, and adjusted to the desired bacterial density. The concentration of bacterial suspensions was verified by serial dilution and CFU enumeration on sheep blood agar plates under microaerophilic conditions. Prior to infection, larvae were acclimated to room temperature without food for 24 h. For each experimental group, 10 larvae were placed in sterile Petri dishes and surface-sterilized with 70% ethanol. Using a Hamilton syringe, 20 μL of bacterial suspension containing 1 × 10^7^ CFU was injected into the hemocoel through the last left proleg of each larva. Control larvae were injected with 20 μL sterile PBS. Following infection, larvae were maintained without food and incubated at 37 °C under standard aerobic conditions in the dark. Larval survival was monitored daily for 4 days. Larval survival was assessed by responsiveness to physical stimulation and melanization. Larvae were considered dead when they failed to respond to touch and exhibited extensive melanization. Survival data were recorded daily and used to generate Kaplan–Meier survival curves for comparison among experimental groups. For bacterial burden determination, larvae were injected with approximately 1 × 10^7^ CFU per larva as described above, and hemolymph samples were collected from surviving larvae at 0, 12, 24, 48, and 72 h post-infection. Independent groups of infected larvae were used for bacterial burden determination at each sampling time. Briefly, 20 μL of hemolymph was collected from individual larvae and transferred into sterile 1.5-mL microcentrifuge tubes. Samples were serially diluted 10-fold in PBS to lyse hemocytes and release intracellular bacteria, plated onto sheep blood agar plates, and incubated under microaerophilic conditions at 37 °C for 72 h. Viable bacterial counts were subsequently determined by CFU enumeration and expressed as CFU per larva. Larvae that had died before the designated sampling time were not included in the bacterial burden analysis because standardized hemolymph collection could not be reliably performed. Each experiment was independently performed at least three times using separate bacterial cultures and different batches of larvae. Data were analyzed using the log-rank (Mantel–Cox) test for survival analyses. Differences were considered statistically significant at *P* < 0.05.

### 2.13 Quantification of biofilm formation by crystal violet staining

Flagella-mediated motility contributes to host colonization and has been implicated in *H. pylori* biofilm formation (Hathroubi et al., 2018b). Because *H. pylori* strain 26695 is non-motile and lacks functional flagella (Hoffman et al., 2003), the effect of HP1581 disruption on biofilm formation was evaluated using the motile strain G27. Biofilm formation was quantified using a crystal violet staining assay as previously described with minor modifications (Hathroubi et al., 2020). Briefly, *H. pylori* strains were cultured in Brucella broth under microaerophilic conditions for 36 h and diluted to an OD_600_ of 0.15. Aliquots (200 μL) of bacterial suspension were dispensed into sterile 96-well flat-bottom polystyrene microtiter plates (Costar) and incubated statically under microaerophilic conditions at 37 °C to allow biofilm development. Biofilm formation was quantified after 1, 2, 3, and 6 days of incubation. At each time point, planktonic cells were carefully removed by aspiration, and the wells were gently washed twice with 1 × PBS to remove non-adherent bacteria. The remaining biofilms were stained with 200 μL of 0.1% (w/v) crystal violet solution for 2 min at room temperature. Excess stain was removed by aspiration, and the wells were washed with PBS to eliminate unbound dye. Plates were subsequently air-dried for 20 min. The bound crystal violet was solubilized with 200 μL of 70% ethanol, and biofilm biomass was quantified by measuring absorbance at 590 nm using a microplate reader. Higher OD_590_ values were interpreted as increased biofilm biomass.

### 2.14 Analysis of biofilm matrix composition by enzymatic dispersion

The composition of the biofilm extracellular matrix was evaluated by enzymatic dispersion as previously described with minor modifications (Hathroubi et al., 2018b). Biofilms formed by *H. pylori* G27 and its derivative mutant strains were established in sterile 96-well flat-bottom polystyrene microtiter plates under the biofilm-forming conditions described above. Briefly, bacterial cultures were incubated under static microaerophilic conditions for 3 days to allow mature biofilm formation. The culture medium was then carefully aspirated and replaced with fresh Brucella broth containing either proteinase K (10–200 μg/mL) or DNase I (10–100 μg/mL; Sigma-Aldrich). Wells receiving fresh medium without enzyme supplementation served as controls. Plates were subsequently incubated under microaerophilic conditions for an additional 24 h. Following enzymatic treatment, planktonic cells were removed by aspiration, and the remaining biofilms were gently washed with PBS. Biofilms were stained with 0.1% (w/v) crystal violet, and the bound dye was solubilized with 70% ethanol as described in the biofilm formation assay. Biofilm biomass was quantified by measuring absorbance at 590 nm using a microplate reader. The extent of biofilm dispersion was determined by comparing the residual biofilm biomass of enzyme-treated samples with that of untreated control biofilms.

### 2.15 Swimming motility assay

As noted above, the effect of *HP1581* disruption on bacterial motility was evaluated using the motile strain G27. Swimming motility assays were performed as previously described (Asakura et al., 2010) with minor modifications. Briefly, *H. pylori* strains were cultured in Brucella broth under microaerophilic conditions for 36–48 h until reaching an OD_600_ of 1.0 (approximately 1 × 10^8^ CFU/mL). Bacterial cells were harvested by centrifugation (4,000 × g, 10 min, 4 °C), washed, and resuspended in 1 mL of 1 × PBS. A 5-μL aliquot of each bacterial suspension was inoculated onto the center of Brucella soft agar plates containing 0.4% agar and 10% FBS. The plates were incubated under static microaerophilic conditions at 37 °C for up to 6 days. Colony diameters were measured daily, and swimming motility was assessed based on the radial expansion of bacterial growth from the inoculation site. Larger colony diameters were interpreted as increased swimming motility.

### 2.16 Statistical analysis

Statistical analyses were performed using GraphPad Prism version 8.3.0.538 (GraphPad Software, San Diego, CA, USA). Unless otherwise indicated, all experiments were independently performed at least three times, and data are presented as the mean ± standard deviation (SD). For comparisons between two groups, statistical significance was determined using an unpaired two-tailed Student's *t*-test. Survival curves generated from the *G. mellonella* infection model were analyzed using the Kaplan–Meier method, and differences between groups were assessed using the log-rank (Mantel–Cox) test. A *P* value of less than 0.05 was considered statistically significant. Statistical significance was denoted as follows: ^*^P < 0.05, ^**^P < 0.01, ^***^P < 0.001, and ^****^P < 0.0001.

## 3 Results

### 3.1 Sequence and structural characterization of HP1581 in the *H. pylori* strain 26695

To gain insight into the potential function of HP1581, its genetic organization, sequence conservation, and predicted structural features were analyzed. According to the National Center for Biotechnology Information (NCBI) database, the *HP1581* coding sequence is located on the minus strand of the *H. pylori* 26695 chromosome between nucleotides 1,659,473 and 1,660,483 and is flanked by the *HP1580* and *HP1582* genes (Supplementary Figure 1A). Conserved domain analysis revealed that HP1581 contains two characteristic functional domains, including a GT-MraY-like superfamily domain and an Rfe domain (Supplementary Figure 1B). These domains are commonly associated with UDP-*N*-acetylglucosamine-1-phosphate transferases involved in bacterial cell envelope biogenesis. The GT-MraY-like domain is predicted to catalyze the formation of a phosphodiester linkage between undecaprenyl phosphate (Und-P) and N-acetylglucosamine-1-phosphate (GlcNAc-1-P; Al-Dabbagh et al., 2008), whereas the Rfe domain is associated with the initiation of glycoconjugate biosynthesis (Rush et al., 1997; Price and Momany, 2005).

To assess evolutionary conservation, the amino acid sequences of HP1581 from strains 26695 and G27 were aligned with homologous proteins from four Gram-negative bacterial species using Sequence Manipulation Suite 3 (SMS3), version 0.0.1. The HP1581 proteins of strains 26695 and G27 share 96% identities and 98% similarity in amino acid sequence, supporting the use of both strains in the present study while indicating that the observed phenotypic differences between strain backgrounds are unlikely to be attributable to sequence variation in HP1581. Although the overall sequence identity ranged from 24% to 28%, HP1581 shared moderate sequence similarity (45–48%) with its homologs (Supplementary Figure 2A and Table 3), suggesting conservation of key structural and functional features across diverse bacterial species. In addition, transmembrane topology prediction using TMPred identified 11 putative transmembrane helices distributed throughout the protein sequence (Supplementary Figure 2B), indicating that HP1581 is an integral membrane protein. Collectively, these bioinformatic analyses support the annotation of HP1581 as a WecA-like undecaprenyl-phosphate GlcNAc-1-phosphate transferase localized to the inner membrane and predict that it catalyzes the initial step of O-antigen biosynthesis by transferring GlcNAc-1-phosphate from UDP-GlcNAc to Und-P, thereby generating the UndPP-GlcNAc intermediate required for subsequent glycan assembly. These findings provide the bioinformatic rationale for the subsequent genetic and biochemical characterization of HP1581 presented in this study.

**Table 3:** Sequence comparison of *H. pylori* HP1581 protein from *H. pylori* wild-type strain 26695 with the corresponding homologs from other Gram-negative bacteria.

Protein	Species	UniProtKB AC^a^	Identities (%)^b^	Similarity (%)^b^	Length
HP1581 (WecA)	• *Helicobacter pylori* 26695 • *Helicobacter pylori* G27 • *Pseudomonas aeruginosa* PAO1 • *Escherichia coli* K-12 • *Salmonella typhimurium* LT2 • *Neisseria meningitidis* NM3147	• O26101 • B5Z9L1 • Q51364 • P0AC78 • Q9L6R7 • R0UWK3^*c*^	• 100 • 96 • 28 • 26 • 24 • 27	• 100 • 98 • 48 • 45 • 45 • 48	• 336 aa • 333 aa • 303 aa • 367 aa • 367 aa • 239 aa

^a^An accession number (AC) that is assigned to each sequence upon inclusion into UniProt (https://www.uniprot.org/) accessed on 5 March 2025; aa, amino acid.

^b^Identities and similarity were obtained from the BLAST program (https://blast.ncbi.nlm.nih.gov/Blast.cgi).

^c^The original UniProtKB accession R0UWK3 has since become obsolete. The corresponding archived UniParc identifier is UPI00032EAFC5.

### 3.2 HP1581 is required for O-antigen biosynthesis and Lewis antigen expression in the *H. pylori* strain 26695

To investigate the role of HP1581 (WecA) in *H. pylori* LPS biosynthesis, an *HP1581* knockout mutant was generated by homologous recombination and subsequently complemented with a functional copy of the gene in strain 26695. Successful gene disruption and complementation were confirmed by PCR analysis and sequencing (data not shown).

**Figure 2:**
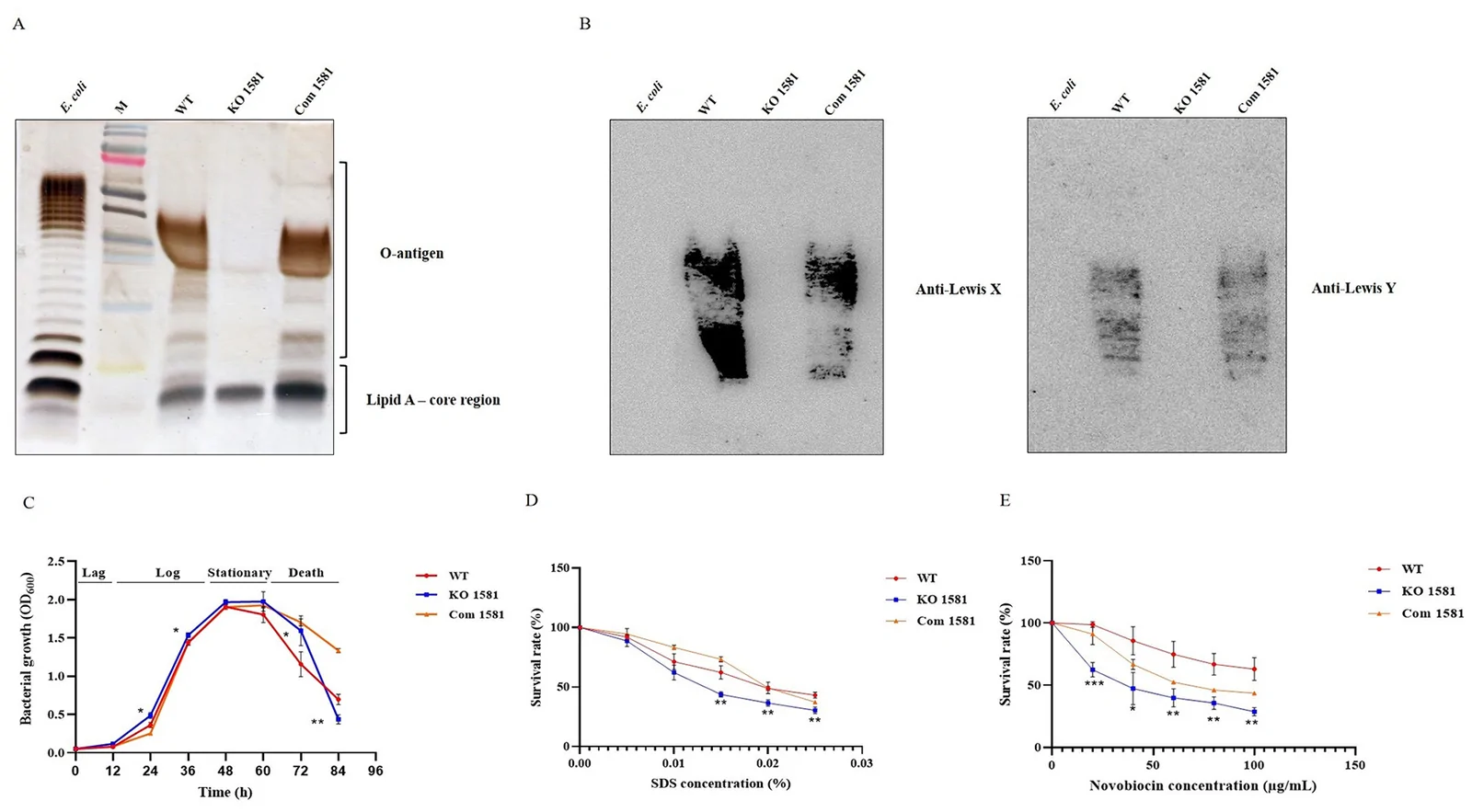
Effects of *HP1581* disruption on LPS structure, Lewis antigen expression, growth, and susceptibility to SDS and novobiocin in the *H. pylori* strain 26695. **(A)** LPS profiles of the wild-type (WT) *H. pylori* 26695 strain, the *HP1581* knockout mutant (KO), and the complemented strain (Com) were analyzed by 15% Tricine-SDS-PAGE followed by silver staining. **(B)** Expression of Lewis X and Lewis Y antigens was examined by immunoblotting using anti- Lewis X and anti- Lewis Y antibodies. The positions of the O-antigen and lipid A-core regions are indicated. **(C)** Growth kinetics of the WT, KO, and Com strains were monitored by measuring optical density at 600 nm (OD_600_) at the indicated time points. Data represent the mean ± SD from three independent experiments. **(D)** Sensitivity to SDS and **(E)** susceptibility to novobiocin were evaluated by determining bacterial survival following exposure to the indicated concentrations of each compound. Survival was calculated as the ratio of the OD_600_ of treated cultures to that of untreated controls. Data represent the mean ± SD from three independent experiments. Statistical significance was determined using an unpaired two-tailed Student's *t*-test (*p < 0.05, **p < 0.01, ****p < 0.001).

To determine whether HP1581 participates in O-antigen biosynthesis, LPS was isolated from the WT 26695, *HP1581* knockout 26695, and complemented 26695 strains and analyzed by Tricine-SDS-PAGE followed by silver staining. The WT and complemented strains exhibited the characteristic full-length LPS pattern with O-antigen-containing molecules, whereas the *HP1581* knockout mutant displayed a markedly truncated LPS profile consisting primarily of lipid A-core structures and lacking the high-molecular-weight O-antigen region (Figure 2A). These findings indicate that HP1581 is essential for O-antigen biosynthesis in *H. pylori*. Because Lewis antigens are major components of the *H. pylori* O-antigen, we next examined Lewis X and Lewis Y expression by immunoblotting using specific monoclonal antibodies. Strong Lewis X and Lewis Y signals were detected in the WT 26695 and complemented 26695 strains, whereas both antigens were completely absent in the *HP1581* knockout mutant (Figure 2B). The loss of Lewis antigen expression is consistent with the absence of O-antigen structures observed by silver staining and further supports a critical role for HP1581 in O-antigen assembly. Collectively, these results demonstrate that HP1581 is indispensable for the biosynthesis of the O-antigen region of *H. pylori* LPS. Disruption of *HP1581* abolishes Lewis antigen expression and results in the production of a truncated LPS structure composed predominantly of lipid A and core oligosaccharide, whereas complementation restores both O-antigen synthesis and Lewis antigen display.

### 3.3 HP1581 contributes to cell envelope integrity and stress resistance in the *H. pylori* strain 26695

To determine whether disruption of HP1581 affects bacterial growth and cell envelope stability, the growth characteristics of the WT 26695, *HP1581* knockout 26695, and complemented 26695 strains were compared under standard culture conditions. As shown in Figure 2C, all three strains exhibited similar growth kinetics during the exponential and stationary phases, indicating that *HP1581* is not essential for bacterial proliferation under laboratory conditions. However, during the death phase, the *HP1581* knockout mutant displayed a more pronounced decline in cell density than either the WT or complemented strain, suggesting reduced survival during prolonged culture.

Because LPS is a major determinant of outer membrane integrity, we next examined the strains' susceptibility to the membrane-disrupting detergent SDS. In the absence of SDS, all strains grew comparably. However, exposure to increasing concentrations of SDS revealed a marked growth defect in the *HP1581* knockout 26695 mutant compared with the WT 26695 and complemented 26695 strains (Figure 2D). The mutant exhibited significantly increased sensitivity even at low SDS concentrations, indicating that loss of HP1581 compromises the outer membrane's protective barrier function. To further assess membrane-associated stress resistance, bacterial susceptibility to the hydrophobic antibiotic novobiocin was evaluated. As shown in Figure 2E, the *HP1581* knockout 26695 mutant displayed substantially reduced growth in the presence of novobiocin relative to the WT and complemented strains. The increased susceptibility to novobiocin is consistent with enhanced membrane permeability resulting from O-antigen deficiency and LPS truncation.

Together, these results demonstrate that disruption of *HP1581* has only a modest effect on bacterial growth under standard culture conditions but significantly impairs resistance to detergent and antibiotic stress. These findings support an important role for WecA-dependent O-antigen biosynthesis in maintaining outer membrane integrity and protecting *H. pylori* against environmental challenges.

### 3.4 *HP1581* disruption increases surface hydrophobicity and autoaggregation in the *H. pylori* strain 26695

**Figure 3:**
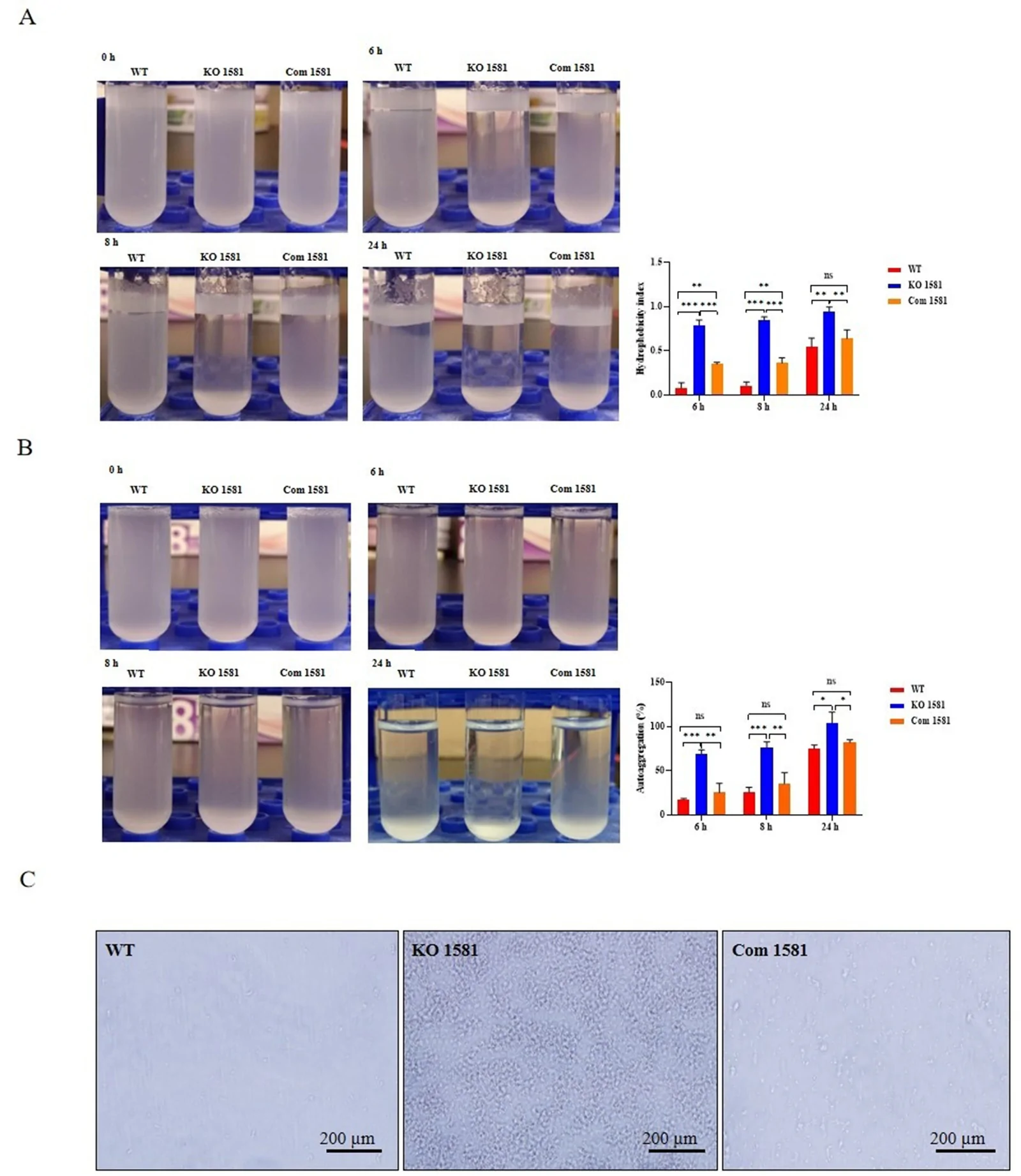
Effects of *HP1581* disruption on surface hydrophobicity and autoaggregation in the *H. pylori* strain 26695. **(A)** Surface hydrophobicity was assessed using an n-hexadecane partitioning assay. Representative images and the corresponding hydrophobicity indices of the wild-type (WT), *HP1581* knockout mutant (KO), and complemented strain (Com) are shown. **(B)** Autoaggregation was evaluated following static incubation in phosphate-buffered saline (PBS). Representative images and the corresponding autoaggregation rates of the WT, KO, and Com strains are shown. The hydrophobicity index and autoaggregation rate were calculated as described in the Materials and Methods. Optical density at 600 nm (OD_600_) at time zero was used as the baseline for calculation. **(C)** Representative microscopic images of bacterial aggregates after 24 h of static incubation. Bacterial clumping was examined using an inverted microscope at 200× magnification. Representative images shown in **(A–C)** are from one of three independent biological experiments. Quantitative data represent the mean ± SD of three independent biological experiments. Statistical significance was determined using an unpaired two-tailed Student's *t*-test by comparing each mutant strain with the WT control (*p < 0.05, **p < 0.01, ***p < 0.001).

To investigate whether loss of WecA affects the physicochemical properties of the bacterial surface, surface hydrophobicity and autoaggregation were compared among the WT 26695, *HP1581* knockout 26695, and complemented 26695 strains. In the n-hexadecane partitioning assay, the *HP1581* knockout 26695 mutant exhibited significantly greater surface hydrophobicity than the WT 26695 strain at all examined time points (6, 8, and 24 h; Figure 3A). Although complementation significantly reduced the mutant's hydrophobicity, the phenotype was not fully restored to WT levels. Because surface hydrophobicity can influence bacterial cell-cell interactions, autoaggregation was subsequently examined under static culture conditions. The *HP1581* knockout 26695 mutant displayed significantly greater autoaggregation across all incubation periods than the WT and complemented strains (Figure 3B). Consistent with these quantitative results, microscopic analysis revealed the formation of larger and more densely packed bacterial aggregates in the mutant strain (Figure 3C). Interestingly, the complemented strain did not fully recover wild-type levels of surface hydrophobicity at 6 and 8 h. Because *HP1581* is transcribed as an independent transcriptional unit rather than as part of an operon, this phenotype is unlikely to result from a polar effect caused by unintended disruption of adjacent genes, suggesting that additional factors beyond simple gene replacement may contribute to the observed defect. Collectively, these findings demonstrate that disruption of *HP1581* significantly alters the surface physicochemical properties of *H. pylori*, resulting in increased surface hydrophobicity and enhanced autoaggregation.

### 3.5 HP1581 disruption alters OMV protein composition and reduces CagA and VacA abundance in the *H. pylori* strain 26695

**Figure 4:**
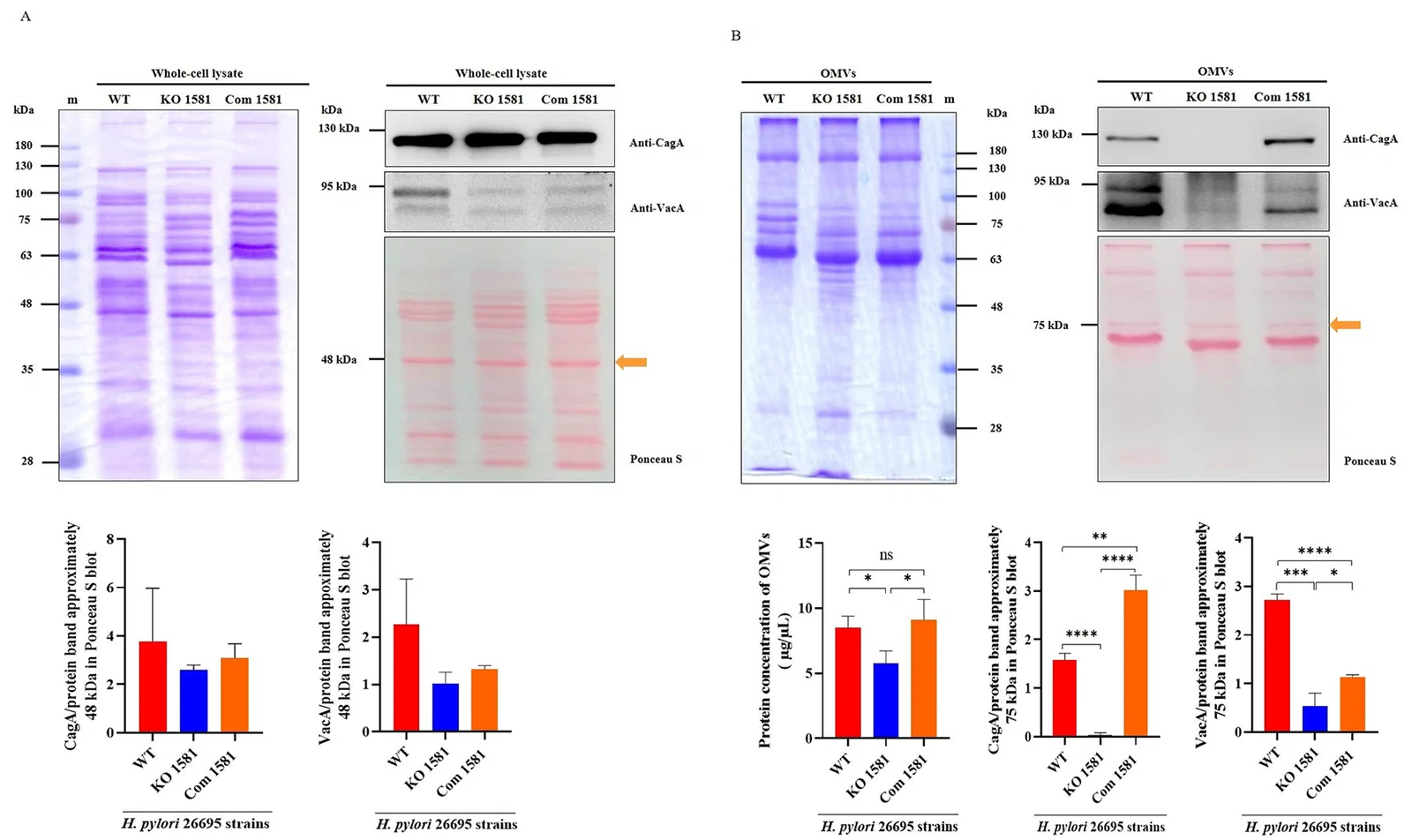
Effects of *HP1581* disruption on CagA and VacA abundance in whole-cell lysates and outer membrane vesicles (OMVs) in the *H. pylori* strain 26695. **(A)** Whole-cell lysates and **(B)** OMVs isolated from the wild-type (WT), *HP1581* knockout mutant (KO), and complemented strain (Com) of *H. pylori* 26695 were analyzed by 10% SDS-PAGE followed by Coomassie blue staining and immunoblotting using anti-CagA and anti-VacA antibodies. Representative Coomassie blue-stained gels, Ponceau S-stained membranes, immunoblots, and their corresponding densitometric analyses are shown. Relative levels of CagA and VacA were quantified from immunoblot signals using ImageJ software. Ponceau S staining was used to verify equal protein loading. The 48-kDa protein band in whole-cell lysates and the 75-kDa protein band in OMV samples (indicated by arrows) were used for normalization of immunoblot signals. Data represent the mean ± SD from three independent experiments. Statistical significance was determined using an unpaired two-tailed Student's *t*-test (*p < 0.05, **p < 0.01, ****p < 0.001, ****p < 0.0001).

To determine whether loss of WecA affects OMV production and cargo composition, whole-cell lysates and OMVs isolated from the WT 26695, *HP1581* knockout 26695, and complemented 26695 strains were analyzed by SDS-PAGE and immunoblotting. Comparison of whole-cell lysates revealed no obvious differences in the overall protein profiles among the three strains (Figure 4A). However, immunoblot analysis showed that the cellular abundance of VacA was reduced in the HP1581 knockout 26695 mutant compared with the WT 26695 strain, whereas CagA levels were only modestly affected (Figure 4A). In contrast, the protein composition of OMVs derived from the *HP1581* knockout 26695 mutant differed markedly from that of the WT strain (Figure 4B), indicating that disruption of HP1581 alters OMV cargo composition. Immunoblot analysis further demonstrated that OMVs isolated from the HP1581 knockout 26695 mutant contained substantially lower levels of both CagA and VacA than those derived from the WT 26695 and complemented 26695 strains (Figure 4B). These results indicate that disruption of HP1581 reduces the cellular abundance of VacA and markedly decreases the levels of both CagA and VacA in OMVs, highlighting a potential role for HP1581-dependent O-antigen biosynthesis in regulating OMV composition and virulence-associated factors.

### 3.6 Disruption of *HP1581* in the *H. pylori* strain 26695 impairs adhesion, internalization, and CagA-dependent responses in AGS cells

**Figure 5:**
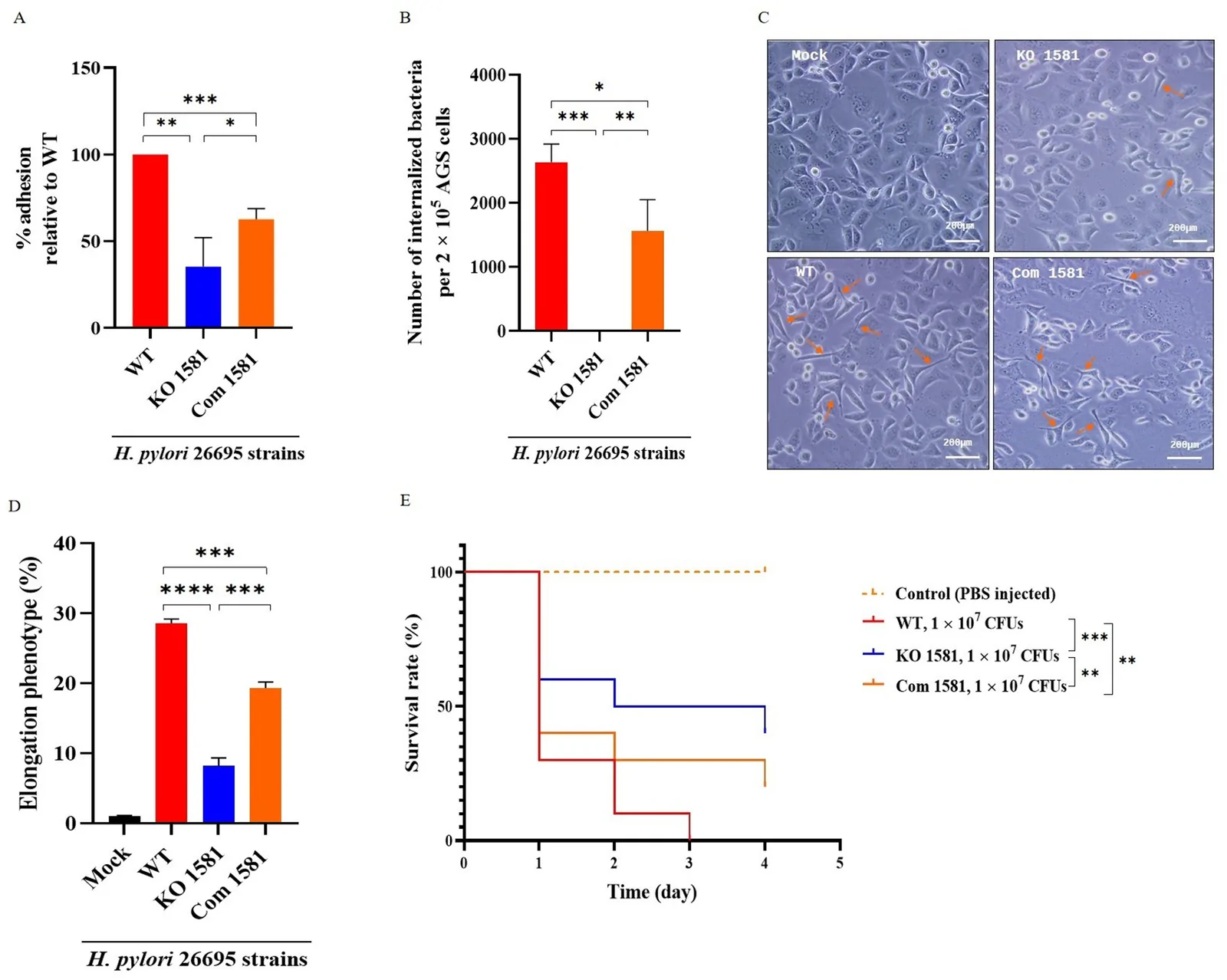
Effects of *HP1581* disruption on host cell interactions and virulence in the *H. pylori* strain 26695. **(A)** Adhesion of the wild-type (WT), *HP1581* knockout mutant (KO), and complemented strain (Com) of *H. pylori* 26695 to AGS gastric epithelial cells was determined following infection at a multiplicity of infection (MOI) of 100. Adherent bacteria were quantified by viable plate counts, and adhesion efficiency was expressed relative to the WT strain. **(B)** Bacterial internalization was assessed under the same infection conditions using a gentamicin protection assay followed by viable plate counts. Internalization efficiency was expressed as the number of intracellular bacteria recovered from 2× 10^5^ infected AGS cells. Data in panels **(A)** and **(B)** represent the mean ± SD from three independent experiments. Statistical significance was determined using an unpaired two-tailed Student's *t*-test (*p < 0.05, **p < 0.01, ***p < 0.001). **(C)** Representative phase-contrast microscopic images of AGS cells infected with the indicated *H. pylori* strains. Cells displaying the elongated hummingbird phenotype are indicated by arrows (scale bar = 200 μm). **(D)** Quantification of the hummingbird phenotype. The percentage of elongated AGS cells was determined by analyzing 10 randomly selected microscopic fields (0.25 mm^2^ per field) from each sample. Data represent the mean ± SD from three independent experiments. Statistical significance was determined using an unpaired two-tailed Student's *t*-test (***p < 0.001, ****p < 0.0001). **(E)** The virulence of *H. pylori* 26695 strains *in vivo* was evaluated using Kaplan–Meier survival analysis in *G. mellonella* larvae following infection with 1 × 10^7^ CFU/larva of either the WT, KO, or Com strains. Each experimental group consisted of 10 larvae, and the survival experiment was independently repeated at least three times using different batches of larvae. Larvae were incubated at 37 °C for up to 4 days, and survival was monitored daily for 4 days. Statistical significance was determined using the log-rank (Mantel–Cox) test (**p < 0.01; ***p < 0.001). PBS, phosphate-buffered saline.

To evaluate the contribution of WecA to host-pathogen interactions, AGS gastric epithelial cells were infected with the WT 26695, *HP1581* knockout 26695, and complemented 26695 strains. Adhesion assays revealed that disruption of *HP1581* significantly reduced bacterial attachment to AGS cells compared with the WT 26695 strain. Complementation significantly increased bacterial adhesion compared with the *HP1581* knockout 26695 mutant, although adhesion remained approximately 37.3% lower than that of the WT strain (Figure 5A). A similar trend was observed in the internalization assay, in which the *HP1581* knockout 26695 mutant showed markedly reduced invasion of AGS cells compared with the WT 26695 and complemented strains (Figure 5B).

To determine whether loss of WecA affects CagA-dependent cellular responses, AGS cell morphology was examined following infection. Cells infected with the WT 26695 and complemented 26695 strains displayed the characteristic elongated hummingbird phenotype, whereas those infected with the *HP1581* knockout 26695 mutant exhibited substantially reduced morphological changes (Figure 5C). Quantitative analysis confirmed a significant reduction in the proportion of elongated cells following infection with the mutant strain (Figure 5D).

Collectively, these results demonstrate that disruption of *HP1581* significantly compromises bacterial adhesion and internalization and attenuates the induction of the elongated (hummingbird) phenotype in AGS cells. These findings underscore the critical role of WecA-dependent O-antigen biosynthesis in mediating host-pathogen interactions and contributing to *H. pylori* virulence.

### 3.7 Disruption of *HP1581* in the *H. pylori* strain 26695 attenuates virulence in the *Galleria mellonella* infection model

To further evaluate the contribution of WecA to *H. pylori* virulence, the *G. mellonella* larval infection model was used as a cost-effective, well-established system for assessing bacterial pathogenicity (Senior and Titball, 2020; Nguyen et al., 2025). Based on our previously optimized infection conditions, larvae were injected with 1 × 10^7^ CFU of the WT 26695, *HP1581* knockout 26695, or complemented 26695 strain and monitored for survival over a 4-day period. As shown in Figure 5E, larvae infected with the *HP1581* knockout 26695 mutant exhibited significantly higher survival rates than those infected with the WT 26695 or complemented 26695 strains. Approximately 60% of larvae infected with the mutant remained alive on day 1 post-infection, with survival gradually declining to 50% on day 2 and 40% on day 4. In contrast, substantially lower survival rates were observed in larvae infected with the WT or complemented strains. No mortality was detected in the PBS-injected control group throughout the experiment.

To assess bacterial persistence within the host, bacterial burden was determined by quantifying viable bacteria recovered from larval hemolymph at 0 (T0), 12, 24, 48, and 72 h post-infection. The initial time point (T0) was set at 30 min after bacterial injection to allow the inoculum to circulate within the larval hemocoel. As summarized in Table 4, all tested *H. pylori* 26695 strains were consistently recovered at T0, confirming successful infection of the larvae. Thereafter, bacterial loads declined progressively in all groups. While the WT 26695 and complemented 26695 strains remained detectable throughout the 72-h observation period, the *HP1581* knockout 26695 mutant could no longer be recovered after 48 h post-infection. No bacterial colonies were detected in PBS-injected control larvae. Together, these results demonstrate that disruption of *HP1581* significantly attenuates *H. pylori* virulence in the *G. mellonella* infection model, as evidenced by increased larval survival and reduced bacterial persistence within the hemocoel.

**Table 4:** Viable counts (CFUs; mean ± SD) of various *H. pylori* 26695 strains in the hemolymph of *G. mellonella* at 0, 12, 24, 48, and 72 h post-infection.

Strains	Ti^a^	T0^b^	T12	T24	T48	T72
PBS (1 × )	0	0	0	0	0	0
WT 26695	2.2 (±0.8) × 10^7^	1.8 (±0.8) × 10^7^	0.8 (±0.2) × 10^6^	3.8 (±0.2) × 10^4^	2.9 (±0.9) × 10^3^	5.0 (±1.0) × 10^1^
KO 1581 (26695)	2.1 (±0.8) × 10^7^	1.4 (±0.4) × 10^7^	2.1 (±0.2) × 10^4^	3.1 (±0.8) × 10^2^	0	0
Com 1581 (26695)	2.4 (±0.5) × 10^7^	1.7 (±0.6) × 10^7^	2.2 (±0.3) × 10^5^	3.0 (±1.0) × 10^3^	1.0 (±0.0) × 10^3^	0.3 (±0.6) × 10^1^

^a^Ti is the original time for larval infection.

^b^Time 0 corresponds to 30 min after larval infection.

### 3.8 O-antigen-deficient mutants exhibit enhanced biofilm formation and reduced motility in the *H. pylori* strain G27

**Figure 6:**
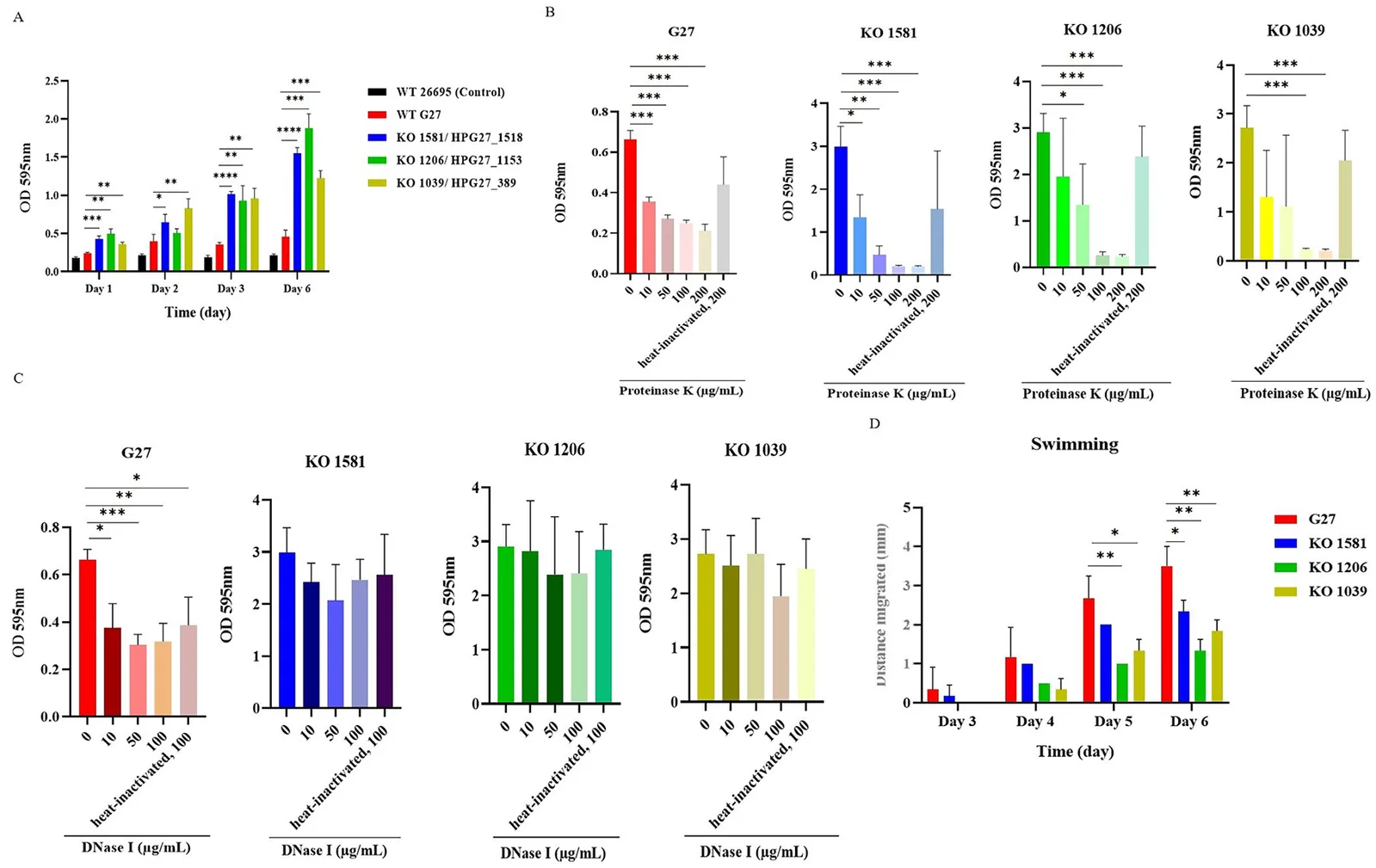
Effects of O-antigen biosynthesis defects on biofilm formation, biofilm stability, and motility in the *H. pylori* strain G27. **(A)** Biofilm formation was quantified using a crystal violet microtiter plate assay. Biofilm biomass of the wild-type (WT) G27 strain and mutants deficient in O-antigen biosynthesis (*HP1581/wecA, HP1206/wzk*, and *HP1039/waaL*) was measured after the indicated incubation periods. Data represent the mean ± SD from three independent experiments, each performed with five technical replicates. Statistical significance was determined using an unpaired two-tailed Student's *t*-test relative to the WT G27 strain (*p < 0.05, **p < 0.01, ***p < 0.001, ****p < 0.0001). The WT 26695 strain was included as a reference control. **(B)** The contribution of extracellular proteins to biofilm stability was evaluated by treating established biofilms with Proteinase K at the indicated concentrations (0, 10, 50, 100, and 200 μg/mL), or heat-inactivated Proteinase K (200 μg/mL). Residual biofilm biomass was quantified after 24 h of incubation at 37 °C under static conditions. Data represent the mean ± SD from three independent experiments. Statistical significance was determined using an unpaired two-tailed Student's *t*-test (*p < 0.05, **p < 0.01, ***p < 0.001). **(C)** The contribution of extracellular DNA (eDNA) to biofilm stability was assessed by treating established biofilms with DNase I at the indicated concentrations (0, 10, 50, and 100 μg/mL), or heat-inactivated DNase I (100 μg/mL), under the same conditions. Residual biofilm biomass was quantified and compared with untreated controls. Data represent the mean ± SD from three independent experiments. Statistical significance was determined using an unpaired two-tailed Student's *t*-test (*p < 0.05, **p < 0.01, ***p < 0.001). **(D)** Swimming motility was evaluated on Brucella broth soft agar (0.4% agar). The corresponding migration distances after 5 days of incubation at 37 °C under microaerobic conditions are shown. Data represent the mean ± SD from three independent experiments. Statistical significance was determined using an unpaired two-tailed Student's *t*-test (*p < 0.05, **p < 0.01). WT, wild-type strain G27; KO, knockout mutant derived from strain G27; Com, complemented mutant derived from strain G27.

Because flagella-mediated motility is an important contributor to the early stages of biofilm development, facilitating bacterial movement toward surfaces, initial attachment, and microcolony formation (Giacomucci et al., 2019), biofilm-related experiments were performed using the motile and flagellated *H. pylori* G27 strain rather than the non-motile strain 26695. To determine whether disruption of O-antigen biosynthesis promotes biofilm formation, biofilm biomass was compared among the WT G27 strain and mutants carrying disruptions in *HP1581* (*wecA*), *HP1206* (*wzk*), or *HP1039* (*waaL*), which represent three sequential steps of the O-antigen biosynthetic pathway, namely O-antigen initiation, translocation, and ligation, respectively. Biofilm biomass was quantified by crystal violet staining after 1, 2, 3, and 6 days of incubation. Compared with the WT G27 strain, which exhibited limited biofilm-forming ability, the *HP1581, HP1206*, and *HP1039* mutants produced significantly greater biofilm biomass (Figure 6A). These findings indicate that disruption of O-antigen biosynthesis, regardless of the biosynthetic step affected, promotes biofilm formation in *H. pylori*.

To identify the major structural components of the biofilm matrix, established biofilms were treated with DNase I or proteinase K. Proteinase K treatment significantly reduced biofilm biomass in all tested strains, whereas DNase I treatment had little or no effect on biofilm stability (Figures 6B, C). These results suggest that extracellular proteins play a more prominent role than extracellular DNA (eDNA) in maintaining the biofilm matrix, consistent with previous observations in *H. pylori* biofilms (Hathroubi et al., 2018b).

Because motility is closely associated with biofilm development, swimming motility was also evaluated. The *HP1581, HP1206*, and *HP1039* mutants derived from the G27 strain exhibited markedly reduced swimming motility compared with the WT G27 strain, with differences becoming particularly evident from day 5 onward (Figure 6D). Despite exhibiting reduced swimming motility, these mutants formed significantly more biofilm than the WT G27 strain, suggesting that enhanced biofilm formation is not necessarily dependent on increased motility and is more likely associated with altered surface properties and enhanced bacterial aggregation resulting from disruption of O-antigen biosynthesis.

## 4 Discussion

Our previous studies established that HP0857, HP0858, HP0859, and HP0860 are essential components of the heptose biosynthetic pathway required for assembly of the LPS core oligosaccharide in *H. pylori* (Chang et al., 2011; Yu et al., 2016; Chiu et al., 2021; Nguyen et al., 2025). However, comparatively little was known about the enzymes involved in O-antigen biosynthesis. Although HP1581 had previously been predicted to encode a WecA-like undecaprenyl-phosphate *N*-acetylglucosamine-1-phosphate transferase based on sequence homology and genome annotation (Hug et al., 2010; Jia et al., 2017), its biological function had not been fully validated in *H. pylori*. In the present study, we combined bioinformatic, genetic, and biochemical approaches to demonstrate that HP1581 is essential for O-antigen biosynthesis. Sequence analysis revealed a high degree of conservation between HP1581 and WecA homologs from other Gram-negative bacteria and identified characteristic conserved domains and multiple transmembrane regions consistent with its predicted function as an inner membrane-associated glycosyltransferase. Functional characterization confirmed that deletion of *HP1581* resulted in the complete loss of the O-antigen region, including the Lewis X and Lewis Y determinants, producing an LPS structure composed solely of lipid A and the core oligosaccharide. These findings provide direct experimental evidence supporting the predicted role of HP1581 as the initiating glycosyltransferase in O-antigen biosynthesis and are consistent with previous observations in *H. pylori* O-antigen-deficient mutants carrying disruptions in *wecA* or *waaL* (Hug et al., 2010).

The loss of O-antigen biosynthesis had broad physiological consequences. Although disruption of *HP1581* did not substantially affect bacterial growth during the exponential phase, mutant survival declined during prolonged culture, suggesting reduced fitness under stressful conditions. Consistent with LPS's established role as a permeability barrier, the *HP1581* knockout mutant displayed increased susceptibility to SDS and novobiocin, indicating compromised outer membrane integrity (Edwards et al., 2000; Nikaido, 2003). Furthermore, loss of the hydrophilic O-antigen layer significantly increased cell surface hydrophobicity and autoaggregation, reflecting major alterations in bacterial surface physicochemical properties. Similar phenotypes have been observed in other Gram-negative bacteria with truncated LPS structures and are generally attributed to increased exposure of hydrophobic outer membrane components following loss of surface polysaccharides.

Alterations in LPS architecture also had profound effects on host-pathogen interactions. The *HP1581* mutant exhibited markedly reduced adhesion to AGS cells, diminished induction of the elongated (hummingbird) phenotype, reduced cellular abundance of VacA, and markedly lower levels of both CagA and VacA in OMVs. Together, these findings suggest that HP1581-dependent O-antigen biosynthesis contributes not only to bacterial attachment but also to the maintenance of virulence-associated factors and OMV composition, thereby influencing the pathogenic potential of *H. pylori*.

Notably, similar defects in adhesion, antimicrobial resistance, and virulence have been reported for mutants lacking enzymes involved in heptose biosynthesis (*HP0857–HP0860*) or fucose biosynthesis (*HP0044* and *HP1275*), indicating that the phenotypes observed here are part of a broader functional network governed by LPS biosynthesis (Chang et al., 2011; Yu et al., 2016; Chiu et al., 2021; Liu et al., 2022b; Nguyen et al., 2025). Collectively, these observations support the concept that both the core oligosaccharide and O-antigen regions are critical for maintaining the structural and functional integrity of the *H. pylori* cell envelope.

The biological significance of WecA was further supported by the *G. mellonella* infection model. Compared with the WT 26695 and complemented 26695 strains, larvae infected with the *HP1581* knockout 26695 mutant exhibited significantly higher survival rates and markedly reduced bacterial persistence within the hemolymph. Although *G. mellonella* lacks an adaptive immune system, its innate immune responses—including phagocytosis, melanization, antimicrobial peptide production, and reactive oxygen species generation—share important functional similarities with mammalian innate immunity (Senior et al., 2011; Hillyer, 2016; Trevijano-Contador and Zaragoza, 2018; Merino et al., 2021). The attenuated virulence of the *HP1581* mutant in this model, therefore, provides *in vivo* evidence that O-antigen biosynthesis contributes substantially to bacterial fitness and persistence during host infection. Although the present study employed a single inoculum optimized for comparative virulence analysis, future studies using one or more sublethal inocula may provide additional insight into bacterial persistence, host immune clearance, and the dynamics of host–pathogen interactions during *H. pylori* infection. Taken together, our findings establish HP1581 as a central determinant of LPS assembly, cell envelope integrity, host interaction, and virulence in *H. pylori*.

Biofilm formation is a major contributor to the chronic persistence and antibiotic tolerance of *H. pylori* infections (Yonezawa et al., 2015). As an important structural component of the biofilm matrix, LPS plays a significant role in biofilm development and organization (Gaddy et al., 2015; Bianchi et al., 2023). In the present study, disruption of *HP1581* (*wecA*), *HP1206* (flippase), or *HP1039* (ligase), all of which are essential for O-antigen biosynthesis, resulted in defective LPS structures and significantly enhanced biofilm formation compared with the WT G27 strain (Figure 6A). Similar phenotypes have been reported in *Shigella flexneri, Escherichia coli*, and *Porphyromonas gingivalis*, where mutations affecting LPS biosynthesis or truncation of surface polysaccharides promote biofilm development (Nakao et al., 2006, 2012; Chai et al., 2023).

A likely explanation for this phenotype is that O-antigen deficiency alters the physicochemical properties of the bacterial surface. Loss of the hydrophilic O-antigen layer increases exposure of underlying hydrophobic outer membrane components, including lipid A, thereby enhancing cell surface hydrophobicity. Increased hydrophobicity can strengthen bacterial adhesion, reduce electrostatic repulsion between neighboring cells, and promote autoaggregation (Bos et al., 1999; Donlan, 2002; Katsikogianni and Missirlis, 2004; Krasowska and Sigler, 2014; Clarke et al., 2023). Because autoaggregation facilitates the formation of multicellular clusters and microcolonies, it represents a critical early step in biofilm establishment (Rickard et al., 2003; Trunk et al., 2018). Consistent with this model, the *HP1581* mutant exhibited increased surface hydrophobicity, enhanced autoaggregation, and elevated biofilm formation, suggesting that O-antigen-dependent modulation of bacterial surface properties contributes directly to biofilm development.

Previous studies have implicated multiple factors in *H. pylori* biofilm formation, including genes involved in fucosylation, flagellar function, outer membrane architecture, and the cag pathogenicity island (Wong et al., 2016; Hathroubi et al., 2018a). In addition, the secreted neutrophil-activating protein NapA is strongly associated with biofilm development, as its expression increases during biofilm maturation and *napA* mutants exhibit reduced biomass and impaired cell aggregation (Haifer et al., 2022). Our biofilm dispersion experiments further demonstrated that extracellular proteins are major structural components of the *H. pylori* biofilm matrix, as proteinase K treatment significantly reduced biofilm biomass, whereas DNase I treatment had minimal effects (Figures 6B, C). These findings indicate that proteinaceous matrix components contribute more substantially to biofilm stability than extracellular DNA (eDNA) under the conditions tested. The limited effect of DNase I may reflect protection of eDNA by extracellular polymeric substances (EPS) or outer membrane vesicles (OMVs), both of which have been reported to shield nucleic acids from enzymatic degradation and to promote biofilm stability through cell–cell interactions and matrix organization (Grande et al., 2015; Zhang et al., 2020). Future studies will therefore investigate the abundance and molecular composition of biofilm-associated OMVs, including their protein, eDNA, and LPS contents, to further elucidate their contribution to biofilm formation in O-antigen-deficient mutants.

Flagellar-mediated motility is a critical virulence trait that enables *H. pylori* to penetrate the gastric mucus layer and establish persistent colonization of the gastric epithelium. The major flagellin proteins FlaA and FlaB are indispensable for motility, and their expression is tightly regulated by factors such as FlbA and the LuxS/autoinducer-2 (AI-2) quorum-sensing system (McGEE et al., 2002; Osaki et al., 2006). Mutations in *flbA* abolish motility and markedly reduce gastric colonization (McGEE et al., 2002), whereas *luxS*-deficient mutants display impaired motility and infectivity (Osaki et al., 2006), indicating that *H. pylori* coordinates flagellar biogenesis and function in response to environmental and population-dependent cues. Consistent with these observations, disruption of *HP1581* significantly reduced swimming motility (Figure 6D), suggesting that proper O-antigen biosynthesis contributes to flagellar function and may thereby influence colonization efficiency and biofilm development. Although the underlying mechanism remains unclear, the reduced motility observed in the *HP1581, HP1206*, and *HP1039* mutants suggests that defects in O-antigen biosynthesis may influence the expression, assembly, or post-translational modification of proteins required for flagellar biogenesis or function. Future studies examining flagellar gene expression, flagellin production, and flagellar ultrastructure in these mutants will help clarify the mechanistic relationship between LPS biosynthesis and motility in *H. pylori*.

Interestingly, despite exhibiting impaired motility, the *HP1581* knockout mutant displayed significantly enhanced biofilm formation. This apparent paradox highlights the complex interplay between motility and biofilm development. While flagella facilitate surface exploration, initial attachment, and microcolony formation (O'Toole and Kolter, 1998; Belas, 2014), biofilm maturation can become increasingly dependent on cell-cell interactions and extracellular matrix production. Transcriptomic analyses have shown that flagellar genes remain highly expressed in *H. pylori* biofilms, and flagellar mutants exhibit reduced biofilm formation (Hathroubi et al., 2018b). Nevertheless, our findings suggest that the increased surface hydrophobicity and autoaggregation resulting from O-antigen deficiency may compensate for reduced motility and promote biofilm development. Similar inverse relationships between motility and biofilm formation have been reported in other Gram-negative bacteria, where LPS truncation simultaneously impairs motility while enhancing aggregation and biofilm formation (Wood et al., 2006; Niba et al., 2007).

Collectively, our data support a model in which HP1581-dependent O-antigen biosynthesis serves as a key determinant of *H. pylori* lifestyle adaptation. Loss of WecA function results in LPS truncation, increased surface hydrophobicity, enhanced autoaggregation, reduced motility, and elevated biofilm formation, thereby shifting the bacterium from a highly motile colonization phenotype toward a more sessile, biofilm-associated persistence phenotype. This transition is accompanied by attenuation of several virulence-associated traits, including reduced adhesion and internalization of gastric epithelial cells, diminished induction of the elongated (hummingbird) phenotype, and decreased virulence in the *G. mellonella* infection model. Together, these findings suggest a trade-off between virulence and persistence, in which *H. pylori* sacrifices efficient host interactions and dissemination in favor of enhanced environmental resilience and long-term survival. Similar transitions from motile virulent states to sessile biofilm-associated lifestyles have been described in numerous bacterial pathogens and are frequently associated with extensive remodeling of the cell envelope and surface glycoconjugates (Hall-Stoodley et al., 2004; Flemming et al., 2016).

In addition to its structural role in maintaining outer membrane integrity, the O-antigen of *H. pylori* LPS has long been implicated in immune evasion through molecular mimicry of host Lewis blood group antigens and modulation of host innate and adaptive immune responses (Moran, 1996; Appelmelk et al., 1996). In the present study, disruption of *HP1581* abolished Lewis X and Lewis Y expression and markedly attenuated bacterial virulence. However, the experimental models employed—including AGS epithelial cells and the *G. mellonella* infection model—were not designed to directly investigate immune evasion mechanisms. Therefore, although our findings demonstrate that HP1581-dependent O-antigen biosynthesis is essential for multiple virulence-associated phenotypes, the contribution of HP1581 to immune evasion remains to be determined. Future studies using mammalian immune cell models and animal infection systems will be necessary to elucidate how loss of O-antigen and Lewis antigen expression influences host immune recognition, immune modulation, and long-term persistence of *H. pylori*.

The findings of this study further underscore the central role of WecA in *H. pylori* survival, virulence, and biofilm formation. Given its essential function in O-antigen biosynthesis and its broad impact on bacterial physiology, HP1581 (WecA) represents a potential target for anti-*H. pylori* therapeutic intervention. However, the enhanced biofilm-forming capacity observed following *HP1581* disruption suggests that inhibition of this pathway may inadvertently promote a biofilm-associated persistence state. Because biofilms confer increased tolerance to environmental stress and antimicrobial treatment (Hall-Stoodley et al., 2004; Hathroubi et al., 2018a; Krzyżek et al., 2020), future studies should evaluate whether pharmacological inhibition of WecA, either alone or in combination with biofilm-disrupting strategies, can effectively suppress bacterial colonization while minimizing the risk of enhanced biofilm-mediated persistence.

## Data Availability

The datasets analyzed in this study are publicly available. Protein sequences used for the comparative sequence alignment were retrieved from the UniProt database under the following accession numbers: *Helicobacter pylori* 26695 (O26101), *H. pylori* G27 (B5Z9L1), *Pseudomonas aeruginosa* PAO1 (Q51364), *Escherichia coli* K-12 (P0AC78), *Salmonella enterica* serovar Typhimurium LT2 (Q9L6R7), and *Neisseria meningitidis* NM3147 (R0UWK3). All other data supporting the findings of this study are included within the article and its Supplementary Material. Further inquiries can be directed to the corresponding author.
