# Supplementary material for: HP1581 (WecA)-dependent O-antigen biosynthesis governs adhesion, virulence, and biofilm dynamics in Helicobacter pylori
Source: Front Microbiol. 2026 Aug 19;17:1909795. doi: 10.3389/fmicb.2026.1909795 (PMC13533946; doi:10.3389/fmicb.2026.1909795)
Supplement: Supplementary file 1 [file Data_Sheet_1.pdf]

Supplementary Material

(A)

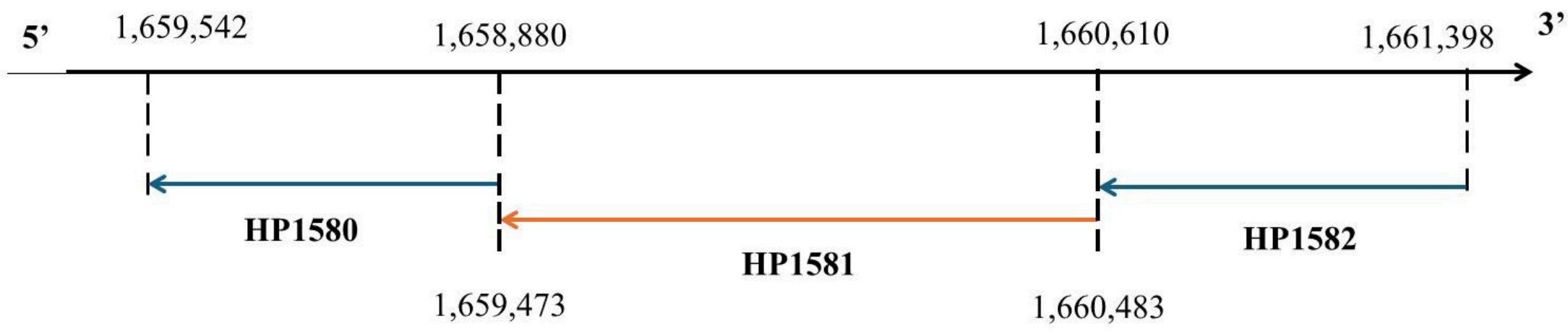

(B)

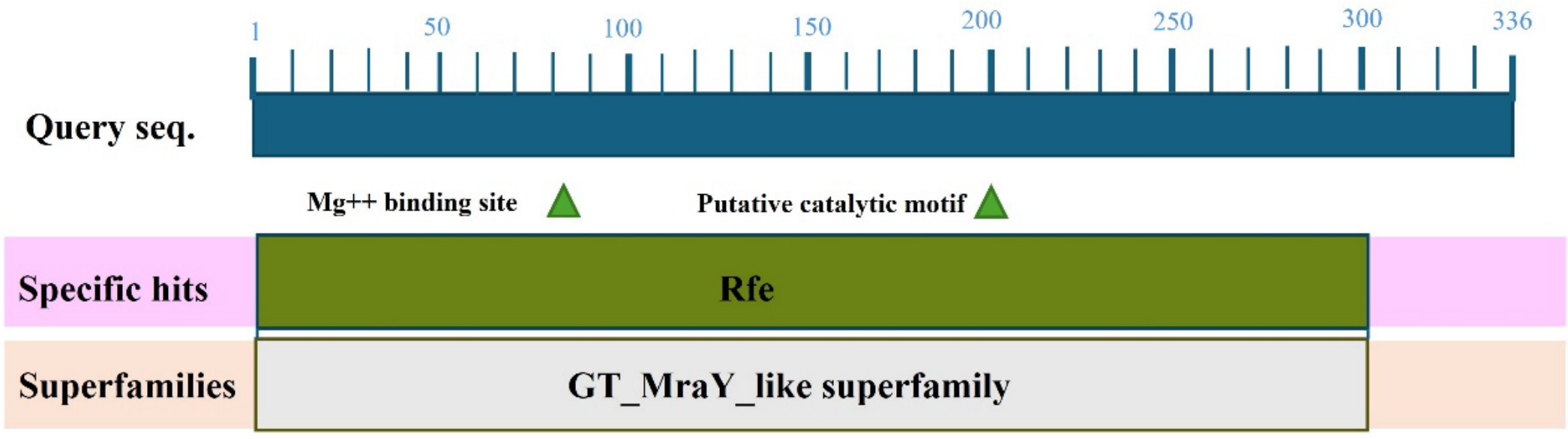

Supplementary Figure 1. Genomic organization and predicted functional domains of HP1581 in *Helicobacter pylori* 26695. (A) Genomic organization of *HP1581* in the *H. pylori* 26695 chromosome. According to the NCBI genome annotation, *HP1581* is located on the negative DNA strand between nucleotide positions 1,659,473 and 1,660,483 and is flanked by the adjacent genes *HP1580* and *HP1582*. (B) Predicted functional domains of the HP1581 protein. Domain analysis identified two conserved domains within HP1581. The GT-MraY-like domain is associated with the transfer of D-*N*-acetylhexosamine-1-phosphate to a membrane-bound polyprenol phosphate acceptor, a key step in bacterial cell envelope biosynthesis and protein glycosylation pathways. The second domain, Rfe, is characteristic of UDP-*N*-acetylglucosamine-1-phosphate transferases and UDP-*N*-acetylmuramyl-pentapeptide phosphotransferases, enzymes involved in the biosynthesis of bacterial cell wall and membrane-associated glycoconjugates. Together, these predicted domains support a role for HP1581 in the initiation of O-antigen biosynthesis.

(A)

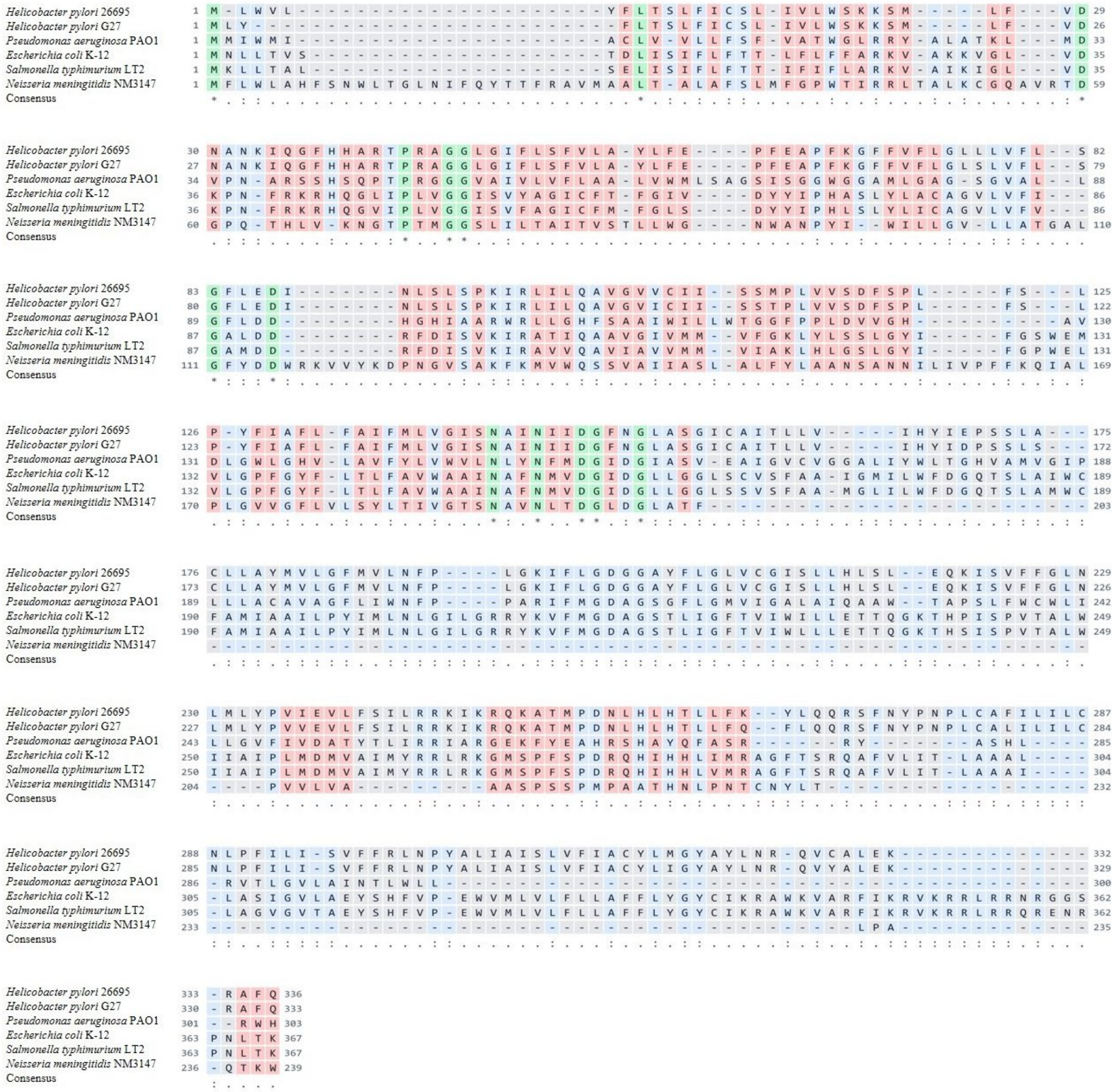

Green fully conserved; blue majority conserved; red variable; gray gap column.
Coordinates count bases or amino acids and ignore gaps.
Consensus; * fully conserved, : majority conserved, . variable.

(B)

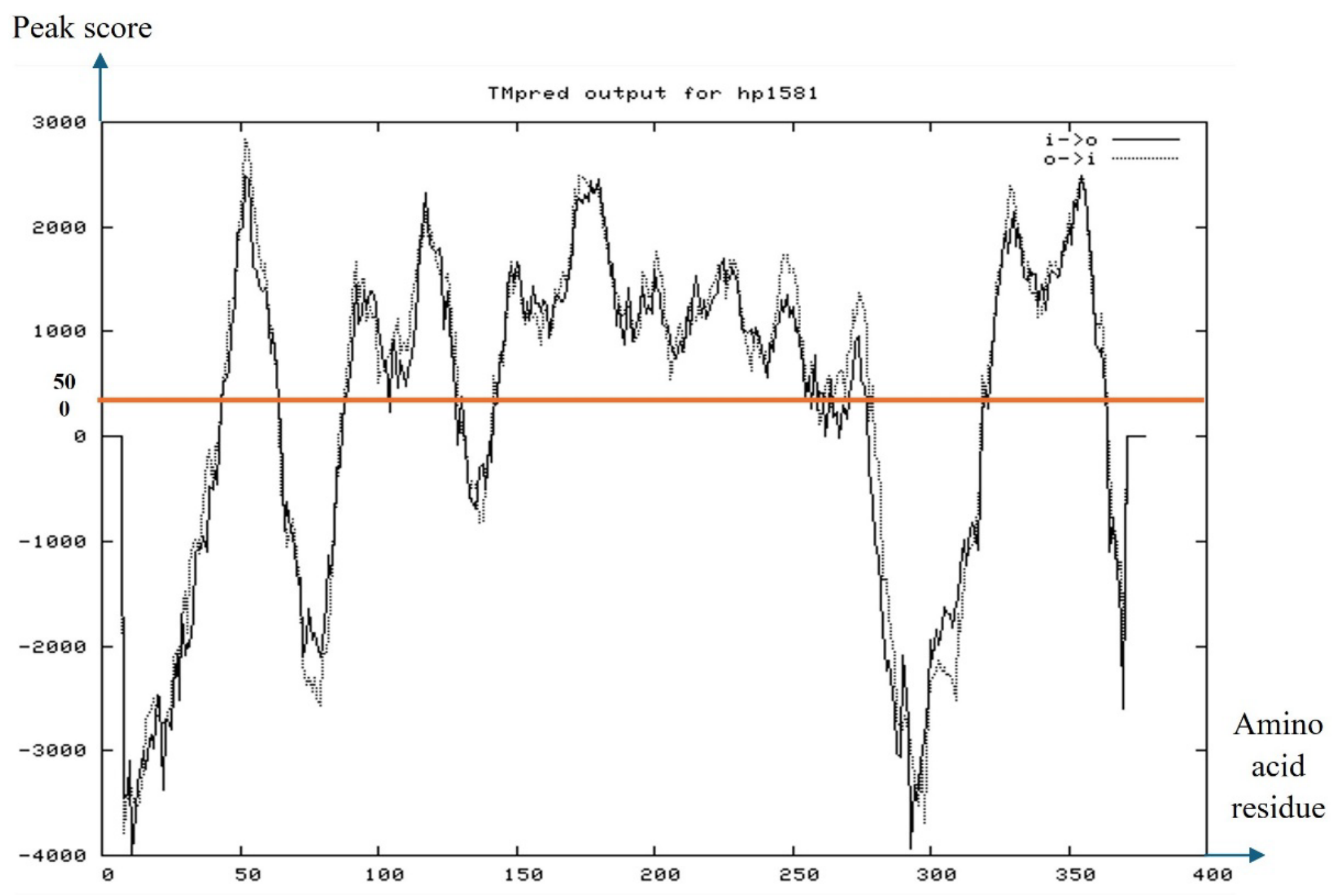

Supplementary Figure 2. Sequence conservation and transmembrane topology analysis of HP1581. (A) Multiple sequence alignment of HP1581 and representative GlcNAc-1-phosphate transferases from diverse Gram-negative bacteria. Amino acid sequences were aligned using Sequence Manipulation Suite 3 (SMS3), version 0.0.1 (https://stothardresearch.ca/sequence-manipulation-suite/#tool=multiple-align-protein). Residue conservation is indicated as follows: green, fully conserved residues; blue, majority-conserved residues; red, variable residues; and gray, alignment gap columns. The aligned protein sequences and their corresponding accession numbers are (from top to bottom): *Helicobacter pylori* 26695 (O26101), *Helicobacter pylori* G27 (B5Z9L1), *Pseudomonas aeruginosa* PAO1 (Q51364), *Escherichia coli* K-12 (P0AC78), *Salmonella enterica* serovar Typhimurium LT2 (Q9L6R7), and *Neisseria meningitidis* NM3147 (R0UWK3). (B) Prediction of transmembrane domains within HP1581 using the TMPred algorithm (http://www.ch.embnet.org/software/TMPRED_form.html). Eleven putative transmembrane helices were predicted between amino acid residues 43 and 365, each exhibiting a prediction score greater than 500. These results indicate that HP1581 is likely an integral inner membrane protein, consistent with its proposed role as a WecA-like UDP-*N*-acetylglucosamine-1-phosphate transferase involved in the initiation of O-antigen biosynthesis.
